# Complete sequencing of *Novosphingobium* sp. PP1Y reveals a biotechnologically meaningful metabolic pattern

**DOI:** 10.1186/1471-2164-15-384

**Published:** 2014-05-19

**Authors:** Valeria D’Argenio, Eugenio Notomista, Mauro Petrillo, Piergiuseppe Cantiello, Valeria Cafaro, Viviana Izzo, Barbara Naso, Luca Cozzuto, Lorenzo Durante, Luca Troncone, Giovanni Paolella, Francesco Salvatore, Alberto Di Donato

**Affiliations:** CEINGE-Biotecnologie Avanzate, Napoli, Italy; Dipartimento di Medicina Molecolare e Biotecnologie Mediche, Università di Napoli Federico II, Napoli, Italy; Dipartimento di Biologia, Università di Napoli Federico II, Napoli, Italy; Dipartimento di Medicina e Chirurgia, Università degli Studi di Salerno, Salerno, Italy; IRCCS-Fondazione SDN, Naples, Italy

**Keywords:** *De novo* sequencing, *Novosphingobium sp*. PP1Y, Sphingomonads, Next generation sequencing, Aromatic pollutant compounds/bioremediation

## Abstract

**Background:**

*Novosphingobium* sp. strain PP1Y is a marine α-proteobacterium adapted to grow at the water/fuel oil interface. It exploits the aromatic fraction of fuel oils as a carbon and energy source. PP1Y is able to grow on a wide range of mono-, poly- and heterocyclic aromatic hydrocarbons. Here, we report the complete functional annotation of the whole *Novosphingobium* genome.

**Results:**

PP1Y genome analysis and its comparison with other *Sphingomonadal* genomes has yielded novel insights into the molecular basis of PP1Y’s phenotypic traits, such as its peculiar ability to encapsulate and degrade the aromatic fraction of fuel oils. In particular, we have identified and dissected several highly specialized metabolic pathways involved in: (i) aromatic hydrocarbon degradation; (ii) resistance to toxic compounds; and (iii) the quorum sensing mechanism.

**Conclusions:**

In summary, the unraveling of the entire PP1Y genome sequence has provided important insight into PP1Y metabolism and, most importantly, has opened new perspectives about the possibility of its manipulation for bioremediation purposes.

**Electronic supplementary material:**

The online version of this article (doi:10.1186/1471-2164-15-384) contains supplementary material, which is available to authorized users.

## Background

Aromatic compounds are among the most widespread dangerous pollutants [1]. Petroleum and its derivatives are the main sources of aromatic molecules released into the environment. The aromatic hydrocarbon content of petroleum can range from about 20% to more than 40% [2–4], whereas the aromatic hydrocarbon content of gasoline and diesel oil is about 30% and 25%, respectively [5, 6].

*Novosphingobium* sp. strain PP1Y is a recently isolated marine α-proteobacterium that is able to grow on a surprisingly wide spectrum of pure mono-, poly- and heterocyclic aromatic hydrocarbons and on complex mixtures of aromatic hydrocarbons dissolved in paraffin oil phases including gasoline and especially diesel-oil which is an optimal growth substrate. Moreover, PP1Y can emulsify diesel-oil by producing small (<1 mm) regular biofilm-covered oil drops that have been described as spherical colonies harbouring a reservoir of growth substrates [7].

Strain PP1Y belongs to the *Sphingomonadaceae* family, which is characterized by the presence of glycosphingolipids in the outer membrane, instead of the more common lipopolysaccharides. This peculiarity renders the surface of their cells more hydrophobic than those of the other Gram-negative strains and, has probably contributed to the development of the ability to degrade mono- and polycyclic aromatic hydrocarbons (PAHs). Moreover, many *Sphingomonadales* harbour several (up to six) large conjugative plasmids, ranging in length from less than 50 kbp to more than 500 kbp [8]. Thanks to these megaplasmids, several *Sphingomonadales* have “collected” genes for the degradation of xenobiotics and continuously exchange them with other bacterial strains [9–11]. Interesting examples are *Novosphingobium aromaticivorans* F199, which uses alkyl-benzenes as the sole carbon and energy source [12], *Novosphingobium pentaromativorans* US6-1, which degrades PAHs with 3–5 aromatic rings [13], *Novosphingobium* sp. TYA-1, which simultaneously degrades bisphenol A and 4-alkylphenols [14]*Sphingomonas paucimobilis* EPA505, which degrades several polycyclic compounds [15], *Sphingomonas wittichii* RW1, which can grow using dibenzofuran and dibenzo-*p*-dioxin [16], *Sphingomonas* sp. TTNP3 which uses alkylphenolic compounds as a source of carbon and energy [17] and *Sphingobium chlorophenolicum* L-1 which degrades pentachlorophenol [11].

Here, we report the analysis of the genome of *Novosphingobium* sp. strain PP1Y and its comparison with the genomes of *N. aromaticivorans* F199 (genome accession number NC_007794.1) [18] and *S. wittichii* RW1 [19], the closest genomes in terms of nucleotide sequence. This comparison has yielded insights into PP1Y and its ability to encapsulate and degrade the aromatic fraction of fuel oils.

## Results and discussion

### Complete genome features and chromosomal architecture

**Figure 1:**
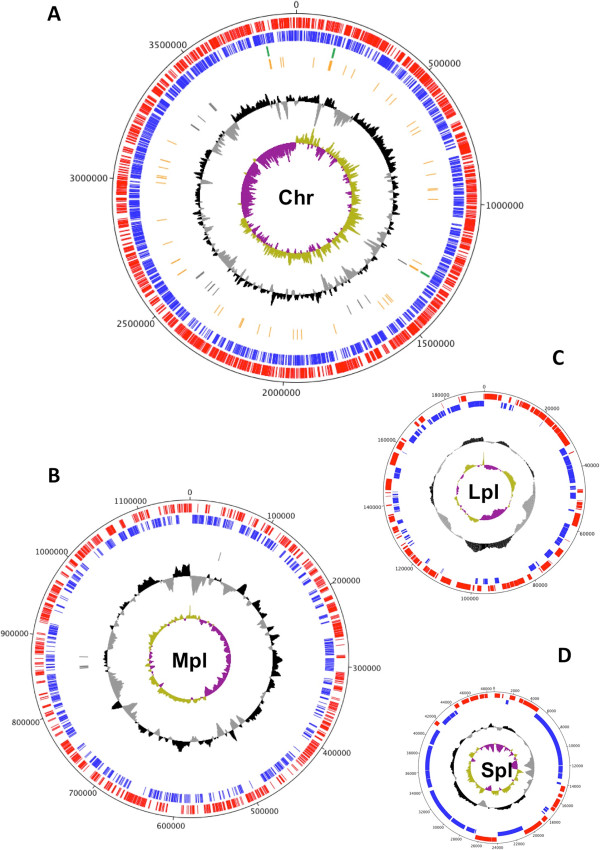
**Circular maps and genetic features of PP1Y replicons.** The principal genomic features of PP1Y Chr **(A)** and its plasmids **(B, C and D)** are shown. For each of them, we report, from outside to the center, genes on the forward and on reverse strands (red and blue), GC content (black) and GC skew (green and violet).

PP1Y genome sequence assembly produced four replicons classified according to their size, as we previously reported [20] (Figure 1A–D). Because the coverage of “small” plasmid (Spl) sequences was, on average, about twice that of the other replicons, it is expected that Spl is present as a two-copy object within each bacterial cell. At present, very few complete sequences of bacterial chromosomes and plasmids are available for organisms of the genus *Novosphingobium* (see Table 1). These sequences have a similar G + C content (about 60%), but PP1Y appears to have the largest and most complex genomic organization of the genus.

**Table 1:** **Principal features and comparison of the genomic sequences available for bacteria of the genus** ***Novosphingobium***

Species	Sequence complete	Sequence available	Size (Mb)	Chrs	Plasmids	Scaffolds	Contigs	CDS	16SrRNA	Identities	Gaps
***Novosphingobium*** **sp. PP1Y**	**yes**	**yes**	**5,073**	**1**	**3**	-	-	**yes**	**yes**	**1,477/1,477**	**0/1,477**
*Novosphingobium* sp. AP12	no	yes	5,611	-	-	-	187	yes	yes	1,437/1,480	6/1,480
*Novosphingobium* sp. Rr 2-17	no	yes	4,539	-	-	-	166	yes	yes	1,424/1,480	6/1,480
*Novosphingobium pentaromativorans* US6-1^a^	no	yes	5,096	-	2	-	123	yes	yes	1,476/1,477	0/1,477
*Sphingobium chlorophenolicum* L-1^b^	yes	yes	4,449	2	1	-	-	yes	yes	1,360/1,480	7/1,480
*Sphingobium indicum* B90A	no	yes	4,082	-	-	-	149	yes	yes	1,373/1,480	7/1,480
*Sphingomonas echinoides* ATCC 14820	no	yes	4,200	-	-	6	64	yes	yes	1,372/1,488	26/1,488
*Sphingomonas wittichii* DP58	no	yes	5,628	-	-	-	739	no	yes	1,372/1,484	16/1,484
*Sphingomonas wittichii* RW1	yes	yes	5,382	1	2	-	-	yes	yes	1,373/1,484	16/1,484
*Novosphingobium aromaticivorans* DSM 12444	yes	yes	3,561	1	2	-	-	yes	yes	1,400/1,480	10/1,480
*Novosphingobium nitrogenifigens* DSM 19370 B057DRAFT	no	yes	4,148	-	-	-	48	yes	yes	1,399/1,478	29/1,478
*Novosphingobium nitrogenifigens* DSM 19370	no	yes	4,140	-	-	-	109	yes	yes	1,400/1,479	31/1,479

^a^no plasmids plA1 and plA2 in the size count; ^b^chr1 + chr2 in the size count.

PP1Y strain is in boldface type.

Various predictive and comparative bioinformatics tools supported by biological databases were used to annotate putative open reading frames (ORFs) and other functional elements [21–27]. As in other bacteria, most of the genome sequence is predicted to be coding and a substantial fraction of predicted ORFs (12-22%, depending on the replicon) appear to have TTG or GTG as the starting codon. Most of them (73% of the 4,709 coding sequences predicted in the four replicons taken together) and all rRNA and tRNA genes are located on the Chr molecule. The same applies to other RNA elements; the only exceptions being three RNAs predicted on Lpl (see Table 2, “Other RNA elements” section and Additional file 1: Table S1).

**Table 2:** **Genome sequencing statistics of the entire PP1Y genome**

	Chr	Mpl	Lpl	Spl
*Genome*				
**Size**, **bp**	3,911,486	1,161,602	192,103	48,714
**G** + **C content, %**	63.7	62.3	60.7	60.1
**Copies for each bacterial cell**	1	1	1	2
*ORFs*				
**Number**	3,481	975	199	54
**Minimum length, bp**	100	100	100	100
**Average length, bp**	990	1,059	788	776
**Maximum length, bp**	7,749	3,597	4,479	5,748
**ATG initiation codons, %**	86.8	88.4	76.4	81.5
**GTG initiation codons, %**	8.8	7.5	12.0	7.4
**TTG initiation codons, %**	4.4	4.1	11.6	11.1
**% of coding sequence**	88.1	88.9	81.6	86.0
*Repetitive elements*				
**Prophage elements**	5	1	1	1
**IS elements**	3	5	7	0
*RNAs*				
**rRNA** **(16S**-**23S**-**5S)**	3	0	0	0
**tRNAs**	58	0	0	0
Other RNA elements*	15	0	3	0
**Cobalamin riboswitches**	5	0	1	0
**suhB**	3	0	0	0
**SRP**_**bact**	1	0	0	0
**Rnase P clA**	1	0	0	0
**tmRNA**	1	0	0	0
**TPP riboswitch**	1	0	0	0
**ctRNA p42d**	1	0	0	0
**Glycine riboswitch**	1	0	0	0
**LR**-**PK1**	1	0	0	0
**GroupII catalytic introns**	0	0	1	0
**ROSE**	0	0	1	0

*Predicted RNA elements, besides tRNAs and rRNAs.

### Evaluation of the putative DNA replication origins

DNA replication was investigated by searching for the putative genome replication origins using a bioinformatic tool. This tool, Orifinder [28], locates predicted bacterial replication origins within each DNA sequence by taking into account base composition asymmetry, distribution of DNA-A boxes and the presence of genes frequently located close to the bacterial replication start (Additional file 1: Table S1). This tool revealed a putative Type-III replication origin on Chr, around base 1, where there is a region of base composition asymmetry containing three DNA-A-boxes, close to the hemE gene (as in the *N. aromaticivorans DSM 12444* genome) and to a DNA-A gene. Differently, on Mpl, Lpl and Spl replicons, Orifinder failed to locate an acceptable putative replication origin, suggesting that other mechanisms may be involved in DNA replications origins. Interestingly, a typical plasmid replication parA/parB/parS cluster was found on each of these replicons, and Mpl and Lpl contain also a predicted plasmid replication repA gene close to the parA/parB/parS cluster, but in a different orientation to those predicted on the *N. aromaticivorans DSM 12444* pNL1 and pNL2 plasmids.

On the Spl plasmid. a complete protein killer gene system is also found, namely, an operon containing two genes that force the host bacteria to retain the plasmid [29].

### Protein genes identified and their significance

**Figure 2:**
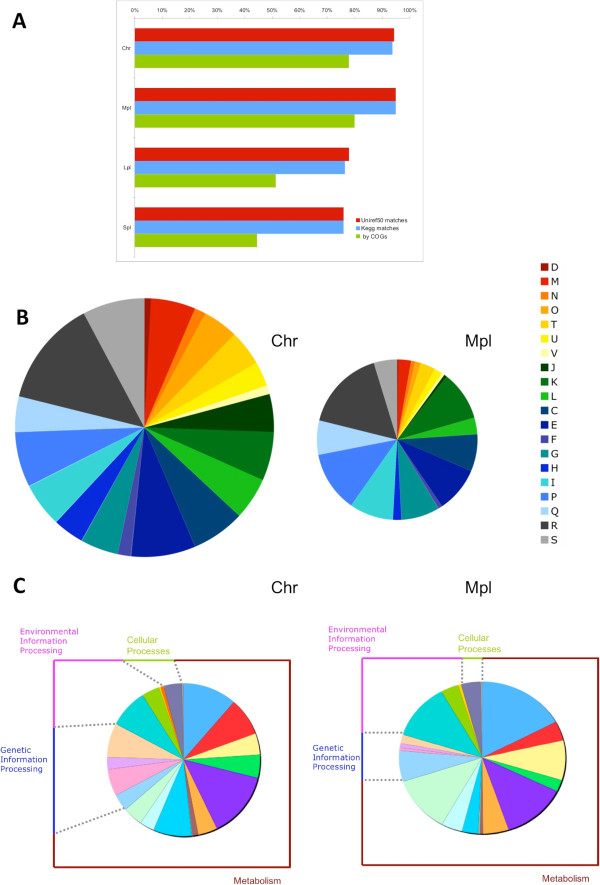
**Open reading frame** **(ORF)** **annotation.** Fraction of ORFs that resemble another protein based on BLAST matches with the Uniref50 and KEGG databases and that have a predicted function according to COG categories **(A)**. The distribution of Chr and Mpl genes is reported with respect to COG functional categories **(B)** and according to Kegg pathways **(C)**. D, Cell cycle control, cell division, chromosome partitioning. M, Cell wall/membrane/envelope biogenesis; N, Cell motility; O, Posttranslational modification, protein turnover, chaperones; T: Signal transduction mechanisms; U: Intracellular trafficking, secretion, and vesicular transport; V, Defense mechanisms; J: Translation, ribosomal structure and biogenesis; K: Transcription; L: Replication, recombination and repair; C, Energy production and conversion; E, Amino acid transport and metabolism; F, Nucleotide transport and metabolism; G, Carbohydrate transport and metabolism; H, Coenzyme transport and metabolism; I, Lipid transport and metabolism; P, Inorganic ion transport and metabolism; Q, Secondary metabolite biosynthesis, transport and catabolism; R, General function prediction only; S. Function unknown.

The gene products encoded by the 4,709 ORFs were characterized by searching for sequence similarity with known bacterial proteins contained in various collections (Figure 2A). About 94% of the ORFs matched at least one protein stored in the Uniref50 or KEGG GENES databases, although the fraction of matched sequences varies and is significantly lower for Lpl and Spl (about 80%). It is noteworthy that about 20% of the ORFs matched proteins annotated as “hypothetical”, “putative” or “uncharacterized”, and are thus classified as coding for “conserved hypothetical proteins”. When the same search was done against protein sequences stored in the COG database, the fraction of identified gene products was lower. In fact, most of the ORFs coding for “conserved hypothetical proteins” did not show any similarity. About 6% of the ORFs did not match any sequence stored in the three databases and are thus classified as coding for “hypothetical proteins”.

An all-against-all comparison of the protein sequences encoded within each replicon was done using BLAST [30] under very stringent conditions (see Table 3, PP1Y-PP1Y section, and Additional file 1: Figure S1A) to look for inter-duplicated genes. About 20% of ORFs from Mpl have a counterpart within the main chromosome (Chr), thereby indicating a partial genome duplication. The whole complement of protein-coding genes was also compared to the one encoded within other complete genomes and plasmids from bacteria of the *Sphingomonadaceae* family (Table 3 and Additional file 1: Figure S1B–D). A number of ORFs ranging between 1,500 and 1,700, i.e. 45-50% of those encoded within Chr in PP1Y, have a counterpart in the main chromosome of the closest analysed bacterial species, the most similar gene set being that from *N. aromaticivorans*, putatively from the same genus. There is no clear evidence that the three smaller replicons are functionally equivalent to other known plasmids in terms of protein coding genes: many protein genes predicted within Mpl appear to have counterparts in *N. aromaticivorans*, although only 20% of Lpl ORFs have a counterpart in the pNL1 plasmid (Accession: NC_009426.1), while others are in the pNL2 plasmid (Accession: NC_009427.1) and some are scattered along the main chromosome. Half the Spl-encoded proteins are encoded by the main chromosome in *N. aromaticivorans. Sphingobium japonicum*[31] and PP1Y share elements of comparable size, although the latter has an additional smaller chromosome. The two species have a 45% similarity within the main chromosome in terms of protein-encoding content, but diverge more extensively in the plasmids. A plasmid from *S. japonicum UT26* pLB1 [32], which is involved in gamma-hexachlorocyclohexane degradation, is somewhat similar to Spl (data not shown).

**Table 3:** **Predicted protein genes comparison**

		Total ORFs	ORFs in PP1Y replicons			
Genome	Replicon		Chr	Mpl	Lpl	Spl
PP1Y	Chr	3,481	3,481	203	30	4
	Mpl	975	240	975	10	-
	Lpl	199	43	10	199	-
	Spl	54	5	-	-	54
*N.aro*	Chr	3,324	1,741	296	43	22
	pNL1	182	203	32	8	-
	pNL2	431	204	232	5	-
*E.lit*	Chr	3,012	1,513	-	-	-
*S.ala*	Chr	3,166	1,536	-	-	-
*S.wit*	Chr	4,851	1,543	-	-	-
*Z.mob1*	Chr	1,801	885	-	-	-
*Z.mobZ*	Chr	1,729	865	-	-	-
*S.jap*	Chr1	3,530	1,573	-	-	-
	Chr2	590	234	147	0	-
	pCHQ1	225	102	11	23	-
	pUT1	45	8	-	-	-
	pUT2	9	-	-	-	-

N. aro = *Novosphingobium aromaticivorans* DSM 12444; E. lit = *Erythrobacter litoralis*; S. ala = *Sphingopyxis alaskenis*; S. wit = *Sphingomonas wittichii*; Z. mob = *Zymomonas mobilis* 11163; Z. mobZ = *Zymomonasmobilis* ZM4; S. jap = *Sphingonium japonicum*.

The protein-coding genes identified in PP1Y are compared with those of several genomes and plasmids.

To assign a putative biological function to protein-coding genes, they were classified, when possible, into COG functional categories based on the result of a BLAST search against COG genes. The predicted protein sequences were also analyzed with the KEGG Automatic Annotation Server KAAS, which assigns a functional annotation to genes following a BLAST alignment against the manually curated KEGG genes database [33] (Additional file 1: Figure S2 A-B). Overall, the Chr sequence of PP1Y contains practically all the core metabolism genes; notably, a number of predicted transporters and transcription factors are present in Mpl (Figure 2B-C).

### Characterization of the PP1Y genes involved in aromatic hydrocarbon degradation

The degradation of aromatic hydrocarbons requires activation of the aromatic ring. This generally occurs by dihydroxylation of the aromatic ring catalyzed by pairs of monooxygenases or dioxygenases/dehydrogenases that constitute the upper pathways. Ring activation is followed by ring cleavage catalyzed by specialized dioxygenases (intra- and extradiol dioxygenases) that start the lower pathways. In the case of methylated aromatic compounds, the initial step can be a monooxygenation reaction of a methyl group followed by oxidation to carboxylate. These reactions can be catalyzed by soluble dioxygenases or by membrane monoxygenases related to xylene monooxygenase XylM. The arylcarboxylate eventually undergoes ring dihydroxylation and cleavage [34]. Analysis of the PP1Y genome revealed at least 81 ORFs (Table 4) that potentially code for the enzymes of both the upper (ring activation) and lower (ring cleavage) pathways.

**Table 4:** **Number of ORFs coding for potential upper (ring activation) and lower (ring cleavage) pathway enzymes in strains PP1Y and F199**

	***Novosphingobium*** sp. PP1Y	***Novosphingobium aromaticivorans*** F199
Ring hydroxylating dioxygenases	38^a^	27
Membrane-bound monooxygenases	2	1
Molybdopteryn-dependent monooxygenases	5	3
Flavine monooxygenases	19	n.d.
2-oxoglutarate-dependent oxygenases (taurine-dioxygenase like)	1	0
2-oxoglutarate-dependent oxygenases	2	1
Extradiol ring-cleavage dioxygenases	10^a^	6
Protocatechuate 4,5 dioxygenases (estradiol ring-cleavage)	4	3

n.d., not determined.

^a^Four ORFs coding for ring hydroxylating dioxygenases and 3 ORFs coding for extradiol ring-cleavage dioxygenases are duplicated, therefore, the genome of PP1Y codes for 34 potential ring hydroxylating dioxygenases and 7 potential extradiol ring-cleavage dioxygenases.

**Figure 3:**
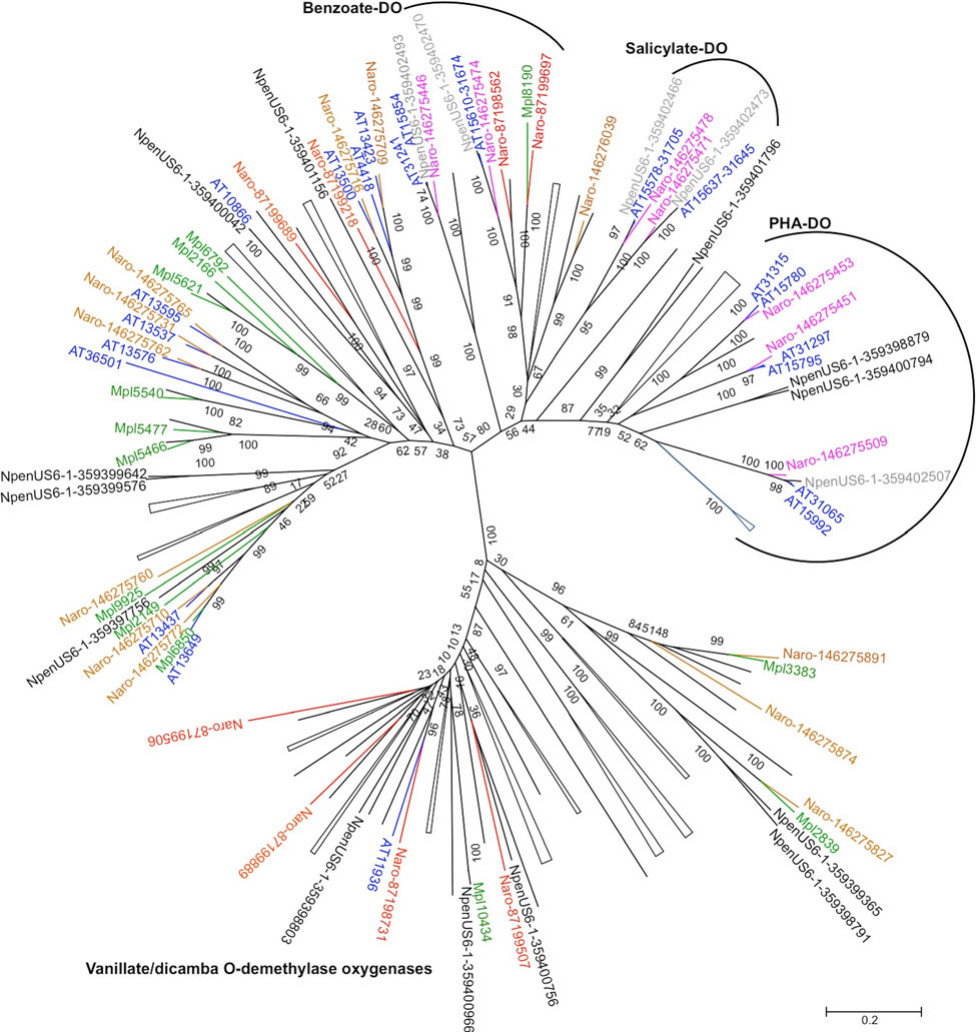
**Neighbor-Joining tree summarizing the relationships among the alpha subunits of the dioxygenases of strains PP1Y, F199 and US6**-**1.** Colours indicate the localization of the ORFs: blue PP1Y/chromosome; green, PP1Y/megaplasmid; red, F199/chromosome; magenta, F199/pNL1; brown, F199/pNL2; black, US6-1/chromosome; gray, US6-1/pLA1. The numbers following the name of the oxygenases refer to the *gi* accession numbers of the NCBI protein database. The analysis involved 164 amino acid sequences (the sequences used to prepare the tree in Additional file 1: Figure S4 plus 18 sequences from strain US6-1). All positions containing gaps were eliminated. There was a total of 150 positions in the final dataset. For clarity all the branches not containing sequences from strains PP1Y, F199 and US6-1 were compressed.

No soluble multicomponent monooxygenase that resembled the well characterized methane monooxygenases and toluene/o-xylene monooxygenase [35] was found in the present study. Thirty-eight ORFs, which were predicted to code for 34 different multicomponent aromatic hydroxylating dioxygenases [36], were identified – a number clearly higher than in the closely related strains *N. aromaticivorans* F199 and *N. pentaromativorans* US6-1 (27 and 18 dioxygenases, respectively) (Figure 3). PPIY has a close counterpart of each F199 dioxygenase: three of these are present in double copy with a 100% identity, which is indicative of a very recent duplication event; and four others have a 90-95% identity, which suggests a less recent duplication event followed by divergence. All duplicated ORFs are closely related to seven ORFs coding for hydroxylating dioxygenases found on plasmid pNL1 from strain F199. Indeed, replicon A of strain PP1Y contains two copies of a region of plasmid pNL1 probably derived by multiple fusion/duplication events (Additional file 1: Figure S3A). Six PP1Y oxygenases from the megaplasmids (Mpl6792, Mpl2166, Mpl5621, Mpl5540, Mpl5477, Mpl5466) do not have homologues in strains F199 and US6-1 but are closely related to predicted oxygenases from strain RW1 (Additional file 1: Figure S4A and B), suggesting that strain PP1Y combined the dioxygenase pools of strains F199 and RW1 and later expanded the pool by duplication events. This strategy enabled PP1Y to expand the pathway for the degradation of naphthalene and methylnaphthalenes, and to degrade larger PAHs. The predicted pathway is shown in Additional file 1: Figure S3B.

Two potential membrane monooxygenases are predicted in PP1Y; they show a 96% identity with each other and a 71-75% identity with the sole membrane monooxygenase found in strain F199, which suggests another recent event of gene duplication. The two PP1Y monooxygenases (Additional file 1: Figure S5) mainly differ in the substrate-binding region, possibly to allow different substrate specificity. No membrane monooxygenase is present in the genomes of *Sphingomonas* sp. MM-1, *Sphingobium japonicum* UT26, *Sphingobium chlorophenolicum* L-1, *Sphingobium* sp. SYK-6, *Sphingobium wittichii* RW1 or *Novosphingobium pentaromativorans* US6-1. This suggests that, also in this case, the PP1Y enzymatic repertoire was expanded by horizontal gene transfer and duplication events.

Ten potential ORFs code for seven homomultimeric extradiol ring cleavage dioxygenases (RCDs) [37], related to *Pseudomonas putida* MT2 catechol 2,3-dioxygenase (Additional file 1: Figure S6). ORF AT15671/AT31616 codes for a putative classic catechol 2,3-dioxygenase; homology modeling and substrate docking have an active site pocket slightly larger than the family benchmark *P. putida* MT2 catechol 2,3-dioxygenase (not shown). AT15599/AT31688 code for a putative dihydroxynaphthalene dioxygenase, and Mpl3065 for a putative 2,3-dihydroxybiphenyl-1,2-dioxygenase. AT32663 is a divergent member of the extradiol dioxygenase family and no closely related sequence is present in the protein databases.

Homology models of these three RCDs are shown in Additional file 1: Figure S7. The four RCDs were cloned, expressed in *Escherichia coli* and their cleavage activity was assayed on 3-methylcatechol, 2,3-dihydroxybiphenyl (2,3-DHBP), and 4-hydroxyoestradiol (4-OHE). The latter was used as analogue of dihydroxy PAHs because these compounds are unstable, difficult to synthesize and not commercially available. The protein coded in AT15599/AT31688 is a very versatile enzyme, able to cleave substrates with 1 to 4 rings (Table 5). Enzyme AT32663 is only active on polycyclic substrates, while Mpl3065 is active only on 2,3-DHBP, as predicted. Finally, AT15671/AT31616 is very active on monocyclic catechols, even though its substrate specificity is wider than that of *P. putida* MT2 catechol 2,3-dioxygenase. Taken together, these four enzymes are able to cleave all classes of 3- and/or 4-substituted catechols in complex mixtures. The other three PP1Y RCDs, AT33026, Mpl10251 and Mpl4329/Mpl4634, are poorly characterized. Preliminary modelling studies suggest that they are dioxygenases specialized in cleaving catechols bearing substituents at positions 3,5 and/or 4,5 and/or 3,6. Therefore, these dioxygenases have a substrate specificity complementary to the four described above.

**Table 5:** **PP1Y RCD specific activity assayed on 3**-**methylcatechol** (**3**-**MC**), **2**,**3**-**dihydroxybiphenyl** (**2**,**3**-**DHBP**), **and 4**-**hydroxy**-**oestradiol** (**4**-**OHE**)

Substrate	Enzyme specific activity (U/mg protein)			
	AT15599/AT31688	Mpl3065	AT32663	AT15671/AT31616
**3**-**MC**	13.54	1.72	0.453	372.9
**2**,**3**-**DHBP**	32.73	12.05	23.5	48
**4**-**OHE**	10.33	0	44.62	3 × 10^-3^

AT numbers refer to the chromosome DNA.

The Neighbor-Joining tree of RCDs (Additional file 1: Figure S6) shows a great heterogeneity among sphingomonads both in the number of potential RCDs (from 1, in the case of strain L-1, to 8 in the case of strain RW1) and in the distribution of the proteins among the RCD subfamilies. Only strains F199 and PP1Y have at least one representative for each subfamily. This particular set of RCDs could allow strain PP1Y to metabolize complex mixtures of catechols deriving from the simultaneous oxidation of several mono- and polycyclic-aromatic hydrocarbons (Additional file 1: Figure S8), which are the preferred substrates for growing this strain.

Besides the seven homomultimeric estradiol RCDs, the PP1Y genome contains also four potential ORFs for heterodimeric extradiol RCDs that are able to cleave catechol rings bearing substituents with carboxylate groups like protocatechuate (see also Additional file 2: Supplementary Results and Discussion). The genome of strain PP1Y contains several other ORFs coding for hypothetical mono- and dioxygenases whose involvement in the degradation of xenobiotics is less clear. Among these, CDS AT10830 is particularly interesting as it codes for a 2-oxoglutarate-dependent oxygenase. These oxygenases cleave different substrates, namely alkyl-sulphonates and fenoxy-acids, by catalyzing monooxygenation reactions of CH bonds adjacent to good leaving groups. Interestingly, no sphingomonad contains a homologous enzyme. Moreover, AT10830 is a member of a group of adjacent ORFs coding for: (i) a hydroxylating dioxygenase (AT10866) that is only distantly related to RW1 and F199 dioxygenases (Additional file 1: Figure S4A); (ii) a heterodimeric extradiol ring cleavage dioxygenase related to 3,4-dihydroxybenzoate dioxygenases; and (iii) a hypothetical acetamidase (AT10838). This cluster of ORFs is present in several distantly related strains including some beta and gamma proteobacteria, thus suggesting a horizontal gene transfer event. At present, nothing is known about the physiological role of this pathway, but its wide diffusion suggests a potentially important ecological role. The data related to Additional file 1: Figures S9–S11 are reported under “Additional file 2: Supplementary Results and Discussion”.

### Stress response genes and their functions

The PP1Y genome contains several ORFs potentially coding for the so-called resistance-nodulation-cell division (RND)-type efflux pumps [38] that actively excrete toxic molecules, and have thus been implicated in the capacity of PP1Y to grow in close contact with a diesel oil phase. (RND)-type efflux pumps are constituted by three subunits: the inner membrane, the outer membrane and the membrane fusion component. The PP1Y genome contains eight potential ORFs for the inner membrane subunit and even more for the other components (Additional file 1: Table S2), suggesting the possible formation of hybrid pumps. The evolutionary relationships among the inner membrane subunits are shown in Additional File 1: Figure S12A.

Three PP1Y RND pumps belong to a subfamily of pumps specific for neutral molecules like aromatic hydrocarbons, acriflavine and other toxic aromatic molecules. The product of AT9347 is closely related to toluene resistance proteins and is very likely an aromatic hydrocarbon resistance protein. Three PP1Y RND pumps belong to a subfamily specific for mono and divalent transition metals and are closely related to a set of RNDs pumps from *Cupriavidus metallidurans* CH34, a benchmark among strains able to tolerate very high concentrations of transition metals [39]. The PP1Y genome also contains eight potential ORFs for P-type ATPases (Additional file 1: Figure S12B), which are membrane ATP-dependent efflux pumps specialized in the excretion of metal cations [40]. For comparison, *C. metallidurans* CH34 genome codes for 9 P-type ATPases.

**Figure 4:**
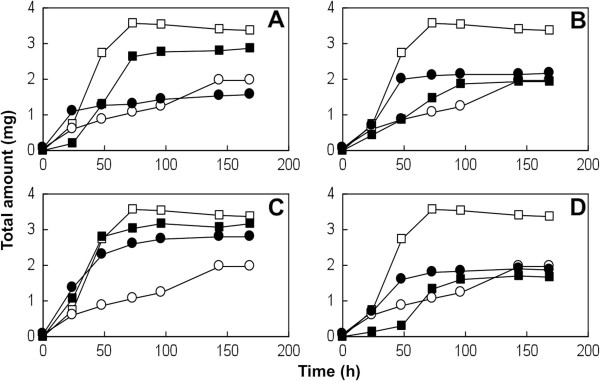
**PP1Y growth**, **measured as total protein and total carbohydrate content in the culture medium, in the presence of millimolar concentrations of heavy metals. (A)** 2.5 mM NiCl_2_; **(B)** 10 mM PbCl_2_; **(C)** 10 mM CuCl_2_; **(D)** 5 mM ZnCl_2_. The control growth shown in all graphs was performed in 1% glutamic acid. Empty squares and circles: total proteins and total carbohydrates, respectively in the control culture. Filled squares and circles: total proteins and total carbohydrates, respectively in the cultures containing metals. Error bars are omitted for clarity; relative error was invariably lower than 8%.

On the basis of these findings, we assayed the ability of PP1Y to grow in liquid medium containing high concentrations of metal cations. Figure 4 shows that PP1Y can grow in the presence of millimolar concentrations of nickel (2.5 mM), lead (10 mM), copper (10 mM) and zinc (5 mM). At higher concentrations, the growth rate steeply decreases to zero (not shown). Interestingly, all the metals increase the carbohydrates/proteins ratio with respect to the control culture, thus suggesting that modification of the cell envelope could contribute to resistance to metals. These results show that the ability of PP1Y to tolerate heavy metals is comparable to that of heavy metal-tolerating strains like *C. metallidurans* CH34 [40], which suggests that PP1Y could play a role in the bioremediation of hydrocarbons in environments polluted by heavy metals.

Tellurite anion is highly toxic to microorganisms (much more than arsenate and arsenite) thanks to its ability to catalyze the oxidation of cell thiols and produce radical oxygen species [41]. Therefore, the wide diffusion of tellurite-resistance mechanisms among bacteria is not surprising, and they might include an aspecific increase of the radical scavenger systems and specific tellurite anion transporters [42]. The PP1Y genome contains three ORFs potentially coding for proteins belonging to three different tellurite-resistance mechanisms: telA (from the *E. coli* kilA/telA/telB system), tehB (from the *E. coli* tehA/tehB system) and terC from *Proteus mirabilis*[43]. Due to the scarce knowledge about these systems, it is difficult to predict their role in tellurite resistance. However, all these ORFs are located in a cluster of ORFs coding for proteins probably involved in detoxification. Interestingly, a similar cluster of ORFs is present in the genome of strain RW1, but not in other sphingomonads (data not shown).

The importance of glutathione as a radical scavenger and mediator of detoxification systems varies among bacteria. However, several bacteria use glutathione and glutathione-dependent enzymes to detoxify reactive organic compounds (like epoxides), halogenated compounds or alkylhydroperoxides, and reactive oxygen species (ROS) such as oxygen radicals [44].

In addition to genes involved in glutathione synthesis and in the reduction of oxidized glutathione, the PP1Y genome codes for 18 glutathione S-transferases (Additional file 1: Table S3). This number is about double that of *E. coli* and suggests that glutathione could play an important role in detoxification of toxic diesel oil components and of toxic metabolites produced by the oxidation of aromatic hydrocarbons, like epoxides and ROS.

The PP1Y genome also codes for six members of a peculiar family of very small (about 100 amino acids) monooxygenases known as “antibiotic biosynthesis monooxygenases” [45]. These enzymes are the only known monooxygenases not containing any metal or flavin cofactors [46], and that prevalently oxidize phenolic groups to quinines. They are involved in at least two very different physiological processes: (i) the synthesis of the polyketide antibiotics (e.g. the products of ActVA-Orf6 of *Streptomyces coelicolor*), and (ii) the quinol redox cycle (e.g. quinol monooxygenase YgiN from *E. coli*). In particular, *E. coli* YgiN could prevent the accumulation of the semiquinone intermediate formed during the oxidation of quinols to quinones thus minimizing the formation of free radical species [47]. At least some of the six PP1Y antibiotic biosynthesis monooxygenases could have similar functions. However, some of them could be also involved in the synthesis of secondary metabolites. It is noteworthy that PP1Y is able to inhibit the growth of molds (unpublished results), which suggests it secretes antifungal compounds.

### Identification of genes involved in extracellular polymer secretion and biofilm formation

The analysis of the PP1Y genome has revealed potential regulatory mechanisms (quorum sensing, QS) and secretion systems for extracellular polymers, including polysaccharides and poly-gamma-glutamate, which may play a role in the complex “social” behavior of PP1Y, a strain able to form different types of multicellular amorphous aggregates and ordered biofilm (see also Additional file 2: Supplementary Results and Discussion). Quorum sensing is a simple molecular mechanism that results in coordinated behavior in response to cell density [48]. The presence in PP1Y of two QS systems is interesting since they could work simultaneously in response to two different cell densities or, could be activated alternatively under specific conditions. Both possibilities could account for PP1Y’s complex behavior.

Although several ORFs for sphingan synthesis have distantly related homologues in the PP1Y genome (identity <30-40%), a gene cluster similar to those present in other *Sphingomonas* does not exist in PP1Y. Therefore, it is unlikely that PP1Y could produce a sphingan-like polysaccharide. However, several clusters potentially coding for the synthesis of extracellular polysaccharides are distributed among the larger replicons (chromosome and Mpl), as shown in Additional file 1: Table S4, Table S5 and Figure S14A. Lpl contains two regions that are probably involved in the synthesis of exopolysaccharides (Additional file 1: Figure S14B), and are widely distributed among sphingomonads. The closest sequences can be found in *S. japonicum* UT26 with an identity of 70-90% at protein level. Interestingly, the region between these two couples of ORFs in Lpl contains five ORFs coding for hypothetical glycosyl transferases and four ORFs coding for the subunits of an ABC-type polysaccharide transport system with high homology in several sphingomonads (Additional file 1: Figure S15 A-B). Lpl651 is particularly interesting as it codes for a large protein containing three glycosyl transferase-like domains. No other sphingomonad contains a representative of this subfamily of glycosyl transferases that can be found in distantly related bacteria, suggesting another case of horizontal gene transfer. Taken together these findings suggest that Lpl codes for the synthesis and export of one or more capsular polysaccharide(s) that probably contains mannose and rhamnose, like sphingans, but whose structures could differ from those produced by other sphingomonads.

Several biofilm-forming strains secrete cellulose as a matrix component. Lpl from PP1Y shares with *Sphingobium japonicum* UT26 a cluster of ORFs coding for a two-subunit cellulose synthase (Additional file 1: Figure S16A), which implicates Lpl in both biofilm synthesis and remodelling. Another CDS coding for a hypothetical cellulase is located on chromosome (AT36325) not far from a CDS coding for an exo-1,3/1,4-beta-glucanase which could act downstream the cellulase (endo-1,4-beta-glucanase) (Additional file 1: Figure S16B). Interestingly, PP1Y has the largest number of glycosyl hydrolases and glycosyl transferases among sphingomonadales and related groups of alpha proteobacteria (Additional file 1: Table S6).

The PP1Y genome contains three ORFs coding for γ-PGA polymerases (Additional file 1: Figure S16C), which are involved in the synthesis of poly-gamma-glutamate, a strongly anionic homopolymer composed of glutamate residues linked by amide bonds between α-amino and γ-carboxyl groups [49]. This polymer can perform different functions, including the stabilization of the extracellular matrix, glutamate storage and toxic metals binding (Additional file 2: Supplementary Results and Discussion).

## Conclusions

This analysis of the annotated *Novosphingobium sp*. PP1Y genome has revealed peculiar biochemical and biotechnological properties, namely, the metabolic pathways specifically involved in: (i) the degradation of a vocabulary of aromatic hydrocarbons, (ii) the resistance to toxic compounds and (iii) the QS social behavior mechanism. This detailed functional evaluation opens new translational perspectives regarding the possible manipulation of the PP1Y genome for bioremediation purposes. Moreover, the comparison between the enzymatic machinery of PP1Y and those of the other sphingomonads able to degrade environmental pollutants suggests that each sphingomonad has independently evolved its own repertoire of degradative enzymes through a complex combination of vertical heredity, horizontal gene transfers, duplications and rearrangements. This process is still ongoing as demonstrated by the presence of multiple copies of pNL1-like regions at different locations of the PP1Y chromosome. As a consequence, even closely related strains like PP1Y, F199 and US6-1, which belong to the genus *Novosphingobium*, have unique features and adaptations to specific, also polluted, environments. The analysis reported in this paper strongly supports the general belief that sphingomonads are very adaptable bacteria with extraordinary genomic plasticity. It also raises biotechnological perspectives of using sphinomonads in bioremediation processes.

## Methods

### Bacterial growth and DNA extraction

*Novosphingobium* sp. strain PP1Y was routinely grown and genomic DNA was extracted as previously described [7].

### Genome sequencing and assembly

The *de novo* whole-genome shotgun sequencing of *Novosphingobium* sp. PP1Y was carried out as described in a preliminary report (EMBL database under accession numbers: FR 856862, FR 856861, FR 856860 and FR 856859 for Chr, Mpl, Lpl and Spl, respectively) [20].

### Sequence annotation

Sequence annotation includes predicted ORFs, rRNAs, tRNAs and other ncRNAs, identified by using the following tools:

- ORFs were predicted by Grc [20] combined with the Uniref50 [21] and KEGG GENES [22] databases;
- rRNA genes and tRNAs, genes were identified by using RNAmmer [23] and tRNAScan-SE [24] respectively;
- Other predicted ncRNA elements were found by Infernal using the RFAM database records as models [25, 26].

In-house developed pipelines guided the whole annotation process, scheduling and running single applications on a 56-blade cluster. ORFs on chromosome, mega-, large- and small plasmids are identified by a number preceded by “AT”, “Mpl”, “Lpl” and “Spl” respectively. All the PP1Y ORFs and their protein sequences discussed in the text and/or included in the trees are available on the “Gene” database at http://www.ncbi.nlm.nih.gov/gene/.

### Phylogenetic analysis

The sequences included in this study were selected by searching public protein databases with BLAST and PSI-BLAST [50]. Clustal Omega [http://www.ebi.ac.uk/Tools/msa/clustalo/] was used to obtain multiple alignments. Alignments were visualized and examined using JalView [51] and MEGA5.1 [52]. Phylogenetic trees were obtained, visualized and manipulated using MEGA5.1. Bootstrap confidence analysis was performed on 1,000 replicates using the Neighbor-Joining method [53]. The evolutionary distances were computed using the Poisson correction method [54] and were expressed as the number of amino acid substitutions per site. All positions containing gaps and missing data were eliminated.

### Subcloning, expression and activity analysis of RCDs

Open reading frames coding for RCDs were amplified by PCR using genomic DNA as template. Gene sequences were engineered to introduce an NdeI site at the 5’-end and a HindIII site at the 3’-end. PCRs were performed in a total reaction volume of 50 μl, containing 50 ng of genomic DNA, 1 μM of each primer, 0.2 mM dNTPs (Roche, Basel, Switzerland), 1× PCR buffer and 2.5 U of Platinum pfx polymerase from *Pyrococcus* sp. (Invitrogen). The amplification program was optimized as follows: initial denaturation at 95°C for 2 min, amplification for 20 cycles of denaturation at 92°C for 1 min, annealing at 56°C for 1 min, extension at 68°C for 1 min. The amplified fragments cut with NdeI and HindIII were cloned into pET22b (+) expression vector (Novagen) previously cut with the same enzymes. RCDs were expressed in *E. coli* strain BL21(DE3), transformed with the appropriate expression vector, purified by ion-exchange chromatography on Q-Sepharose FF resin and analyzed for quality as described previously [55]. Assays were performed at 25°C in 50 mM Tris/HCl (pH 7.5) in a final volume of 500 μl by spectrophotometric determination of the product of the reaction as described elsewhere [55]. The amount of the products was measured using their extinction coefficients: ϵ _388_ = 13,800 M^-1^ cm^-1^ for the product of 3-methylcatechol (3-MC) [55]; ϵ _434_ = 13,200 M^-1^ cm^-1^ for the product of 2,3-dihydroxybiphenyl (2,3-DHBP) [56]; ϵ _298_ = 9,100 M^-1^ cm^-1^ for the product of 4-hydroxy-oestradiol (4-OHE). One unit of enzyme activity was defined as the amount of enzyme required to form 1 μmol of the product per minute under the assay conditions. Specific activity is given as units per milligram of protein.

Synthesis of 4-OHE was achieved by Dr. Pezzella (Department of Chemistry, University of Naples Federico II) via the o-Iodoxybenzoic acid (IBX)-mediated phenolic oxygenation procedure as previously described [57]. All chemicals were of the highest grade available and were from Amersham Biosciences, Promega, New England Biolabs, Sigma, ABCR GmbH, Fluka, or Applichem. *Escherichia coli* strain BL21 (DE3) and plasmid pET22b (+) were purchased from Novagen (Madison, WI, USA). DNA sequences and oligonucleotide synthesis were performed by Eurofins MWG Operon (Germany).

### Heavy metal resistance

The resistance of bacteria to heavy metals was evaluated by measuring bacterial growth according to Notomista et al. [7] in a minimal medium containing 20 mM MOPS pH 6.9, 100 mM NaCl, 1 g/L NH_4_Cl and 1.0% glutamic acid as sole carbon and energy source, plus trace amounts of four heavy metal salts: NiCl_2_, CuCl_2_, ZnCl_2_, and PbCl_2,_ plus four heavy metal salts: NiCl_2_ (2.5 mM), CuCl_2_ (10 mM), ZnCl_2_ (5 mM), and PbCl_2_ (10 mM) (Sigma–Aldrich, St Louis, MO, USA).

### Availability of supporting data

The following additional data are available with the online version of this paper: Additional file 2, which includes Supplementary Results and Discussion; and Additional file 1, which includes Tables S1 to S6 and Figures S1 to S16. Phylogenetic tree newick files are available online as Additional file 3. PP1Y genomic sequences are available in the EMBL database (http://www.ebi.ac.uk/ena/) under accession numbers: FR 856862, FR 856861, FR 856860 and FR 856859 for Chr, Mpl, Lpl and Spl, respectively (http://www.ebi.ac.uk/ena/data/view/Taxon:Novosphingobium%20sp.%20PP1Y).

## Electronic supplementary material

Additional file 1: **Supplementary Tables and Figures.** (PDF 3 MB)

Additional file 2: **Supplementary Tables and Figures.** (PDF 70 KB)

Additional file 3: **Phylogenetic tree newick files.** (ZIP 12 KB)

## Abbreviations

PAHs: Polycyclic aromatic hydrocarbons
Chr: Chromosome
Mpl: Mega plasmid
Lpl: Large plasmid
Spl: Small plasmid
ORFs: Open reading frames.
