# Supplementary material for: Complete sequencing of Novosphingobium sp. PP1Y reveals a biotechnologically meaningful metabolic pattern
Source: BMC Genomics. 2014 May 19;15(1):384. doi: 10.1186/1471-2164-15-384 (PMC4059872; doi:10.1186/1471-2164-15-384)
Supplement: Supplementary file 1 — Additional file 1: Supplementary Tables and Figures. (PDF 3 MB) [file 12864_2013_6112_MOESM1_ESM.pdf]

Additional file 1

Complete sequencing of *Novosphingobium* sp. PP1Y reveals a biotechnologically meaningful metabolic pattern

Valeria D'Argenio[1,2,*], Eugenio Notomista[3,*], Mauro Petrillo[1,2,*], Piergiuseppe Cantiello[1], Valeria Cafaro[3], Viviana Izzo[4], Barbara Naso[1,2], Luca Cozzuto[1], Lorenzo Durante[3], Luca Troncone[3], Giovanni Paolella[1,2], Francesco Salvatore[1,5,§], Alberto Di Donato[3]

*These authors contributed equally to the work

Corresponding author: Francesco Salvatore
E-mail: salvator@unina.it

[1]CEINGE-Biotecnologie Avanzate, Napoli, Italy.
[2]Dipartimento di Medicina Molecolare e Biotecnologie Mediche, Università di Napoli Federico II; [3]Dipartimento di Biologia, Università di Napoli Federico II;
[4]Dipartimento di Medicina e Chirurgia, Università degli Studi di Salerno;
[5]IRCCS-Fondazione SDN, Naples, Italy

TABLE S1 Replication sites in the four PP1Y replicons.

	OriC/dnaA	*parA/parB/parS*	*repA*
**Chr**	yes/yes	yes/yes/yes	no
**Mpl**	no/no	yes/yes/yes	yes
**Lpl**	no/no	yes/yes/yes	yes
**Spl**	no/no	yes/yes/yes*	no

* Spl contains also a parS/parA/parB/parS operon (see section entitled "Evaluation of the putative DNA replication origins" in the main text under Results and Discussion)

TABLE S2 Complete list of the PP1Y potential ORFs for the three subunits of the RND-type Efflux Pumps.

Inner Mem	Membrane-fusion	Outer Mem	periplasm
AT477	AT501		
		AT26323	
AT26892	AT26745	AT26928	
	AT26450	AT26437	
	AT36239		
		AT37190	
AT9347	AT9368	AT9332	
AT28595	AT28585	AT28618	
AT16571	AT16559	AT16549	
	AT17967	AT17956	
Mpl2516	Mpl2495	Mpl2540	
	Mpl5845		
	Mpl9413	Mpl9442	
AT22882	AT22868	AT22851	AT22908
AT22740	AT22725	AT22714	
	AT21473	AT21444	

TABLE S3 PP1Y genes involved in glutathione metabolism.

ORFs AT4214 and AT30465 code for two glutathione peroxidases that could play a role in the detoxification of alkylhydroperoxides. ORFs AT14455 and AT32855 code for lactoylglutathione lyases that catalyze the condensation of glutathione and methylglyoxal to lactoylglutathione. AT13944 codes for hydroxyacylglutathione hydrolase that catalyzes the hydrolysis of glutathione thioesters regenerating free glutathione. AT25438, AT20884 and AT21125 code for enzymes that catalyze the condensation of glutathione and formaldehyde to the corresponding thioacetale (S-hydroxymethyl glutathione). The adjacent ORFs, AT9397 and AT9409, code for a S-hydroxymethyl glutathione dehydrogenase and a S-formylglutathione hydrolase that convert the thioacetale to glutathione and formate. ORF AT9427, close to AT9397 and AT9409, and ORFs AT21359, AT21382 and AT21411 code for the subunits of a formate dehydrogenase that, by oxidizing formate to CO_2, completes the degradation pathway of formaldehyde.

**AT342 / AT11664 / AT11540 / AT9150 / AT4221 / AT11532 / AT28366 / AT21203 / AT11674 / AT11650 / AT20084 / AT28388 / AT28401 / AT33940 / AT11660**	**glutathione S transferase like (15)**
**AT6822**	**glutathione transferase zeta 1 / Maleylacetoacetate isomerase**
**AT31634 / AT15656 / AT29345**	**glutathione S transferase domain containing**
**AT25438 / AT20884 / AT21125**	**glutathione dependent formaldehyde activating (3)**<br><br>**$R\text{-}S^- + H_2CO$ = $R\text{-}S\text{-}CH_2\text{-}OH$ (thioacetal)**
**AT9397**	**S hydroxymethyl glutathione dehydrogenase /alcohol dehydrogenase**<br><br>**$R\text{-}S\text{-}CH_2\text{-}OH$ = $R\text{-}S\text{-}CH{=}O$ (thioester of formic acid)**
**AT9409**	**S formylglutathione hydrolase**<br><br>**$R\text{-}S\text{-}CH{=}O + H_2O$ = $R\text{-}S^-$ + HCOOH**
**AT4214 / AT30465**	**glutathione peroxidase**<br><br>**$2R\text{-}S^- + R'\text{-}OOH$ = $R\text{-}S\text{-}S\text{-}R + R'\text{-}OH$**
**AT13944**	**hydroxyacylglutathione hydrolase**<br><br>**R-S-CO-R’ = R-SH + HOOC-R’**
**AT14455 / AT32855**	**lactoylglutathione lyase**<br><br>**glutathione + methylglyoxal ⇔ hemithioacetal adduct ⇔ (R)-S-lactoylglutathione**
**AT29766**	**glutathione reductase**
**AT25503**	**glutathione synthetase**

TABLE S4 The four large and five small clusters in PP1Y chromosome A coding for hypothetical glycosyl transferases, synthesis of nucleoside-diphosphate-sugar precursors, polysaccharide polymerases and/or export proteins.

**AT7751**	pssK exopolysaccharide polymerization protein
**AT7758**	polysaccharide biosynthesis protein
**AT7767**	glycosyltransferase involved in LPS biosynthesis like protein
**AT7779**	dolichyl phosphate mannose synthase related protein
**AT7787**	glycosyl transferase family protein
**AT7798**	conserved hypothetical protein
**AT7809**	hexapeptide transferase family protein
**AT7814**	periplasmic protein involved in polysaccharide export/polysaccharide export outer membrane protein
**AT7822**	putative glycosyltransferase
**AT7832**	glycosyltransferase
**AT7845**	new prediction 34
**AT7868**	hypothetical protein/UDPglucose 6 dehydrogenase EC 1.1.1.22
**AT7894**	glycosyl transferase family 2
**AT7905**	ypch01112 glycosyltransferase
**AT7911**	glycosyl transferase family 2
**AT7930**	ATP binding protein of ABC transporter/ATP binding cassette subfamily B bacterial
**AT7948**	UDP glucose 4 epimerase
**AT7958**	NAD dependent epimerase dehydratase family protein/dTDP glucose 4 6 dehydratase EC 4.2.1.46
**AT7968**	glycosyl transferase group 1 family protein putative
**AT7979**	Putative uncharacterized protein
**AT7986**	FAD dependent oxidoreductase/glycerol 3 phosphate dehydrogenase EC 1.1.5.3
**AT7994**	dolichol phosphate mannosyltransferase EC 2.4.1.83
**AT8000**	glycosyl transferase family protein
**AT8011**	glycosyl transferase family protein
**AT8041**	NAD dependent epimerase dehydratase putative
**AT8050**	methyltransferase putative

AT10096	glycosyl transferase group 1
AT10111	putative glycosyltransferase protein
AT10122	putative sugar nucleotide epimerase dehydratase protein
AT10133	putative NDP hexose 3 C methyltransferase protein
AT10144	C methyltransferase
AT10159	lpcA galactosyltransferase protein
AT10170	new prediction 50
AT10175	pssL exopolysaccharide polymerization and or export protein
AT10188	glycosyl transferase family 2
AT10201	cellulose biosynthesis protein CelD
AT10209	glycoside hydrolase family 16
AT10230	protein involved in cellulose biosynthesis CelD like protein
AT10241	beta hydroxylase aspartyl asparaginyl family protein
AT10248	ypch01194 glycosyltransferase
AT10257	new prediction 51
AT10266	glycosyl transferase group 1
AT10272	conserved hypothetical protein
AT10294	conserved hypothetical protein
AT10309	rfbP undecaprenyl phosphate galactosephosphotransferase
AT10321	polysaccharide export outer membrane protein

AT33104	mannosyltransferase
AT33116	conserved hypothetical protein
AT33128	polysaccharide biosynthesis protein putative
AT33142	conserved hypothetical protein
AT33150	new prediction 148
AT33153	exoY succinoglycan exopolysaccharide synthesis protein
AT33162	conserved hypothetical protein
AT33178	polysaccharide pyruvyl transferase

AT21571	polysaccharide export protein<br>Periplasmic protein involved in polysaccharide export
AT21576	lipopolysaccharide biosynthesis<br>Chain length determinant protein ;
AT21592	putative exopolysaccharide biosynthesis protein;
AT21603	conserved hypothetical protein
AT21618	ATPase<br>Type II secretory pathway
AT21629	polysaccharide deacetylase
AT21636	glycosyl transferase group 1
AT21638	conserved hypothetical protein
AT21661	ABC transporter related

AT942	glycosyl transferase group 1
AT955	O antigen polymerase

AT749	Transglycosylase SLT domain protein
AT752	UTP glucose 1 phosphate uridylyltransferase
AT764	UDP N acetylglucosamine 1 carboxyvinyltransferase/<br>UDP-N-acetylglucosamine enolpyruvyl transferase

AT837	sugar transferase
AT851	OstA like protein/<br>lipopolysaccharide export system protein LptA
AT855	lipopolysaccharide export system protein LptC

AT12196	glycosyl transferase family 14
AT12206	polysaccharide biosynthesis protein/<br>Membrane protein involved in the export of O-antigen and teichoic acid
AT12221	glycosyl transferase family 2

AT20178	dolichyl phosphate beta D mannosyltransferase
AT20190	glycosyl transferase family protein<br>Dolichyl-phosphate-mannose-protein O-mannosyl transferase

TABLE S5 Three small clusters of ORFs on megaplasmid B coding for hypothetical glycosyl transferases, synthesis of nucleoside-diphosphate-sugar precursors, polysaccharide polymerases and/or export proteins.

**Mpl1952**	polysaccharide deacetylase
**Mpl1955**	glucose galactose transporter/ fucose permease;
**Mpl1975**	glycosyl transferase group 1
**Mpl1986**	conserved hypothetical protein
**Mpl1995**	conserved hypothetical protein
**Mpl2000**	glycosyl transferase group 1

**Mpl9979**	glycosyl transferase group 1
**Mpl9992**	inner membrane protein YghQ;<br>Membrane protein involved in the export of O-antigen and teichoic acid

TABLE S6 Distribution of glycosyl hydrolases and glycosyl transferases among sphingomonadales and related groups of alpha proteobacteria, including strain PP1Y.

	PP1Y	L-1	UT26	RW1	SYK-6	DSM 12444	RB-22 56
GH	53	32	43	16	21	44	25
GT	57	55	54	53	50	32	15

	ATCC 10988	HTCC 2594					
GH	13	19					
GT	20	22					

GH: glycosyl hydrolases
GT: glycosyl transferases

STRAINS
L-1: *Sphingobium chlorophenolicum*
UT26: *Sphingobium japonicum*
RW1: *Sphingomonas wittichii*
SYK-6: *Sphingobium sp.*
DSM12444: *Novosphngobium aromaticivorans*
RB2256: *Sphingopyxis alaskensis*
ATCC10988: *Zymomonas mobilis mobilis*
HTCC2594: *Erythrobacter litoralis*

Supplementary Figure S1

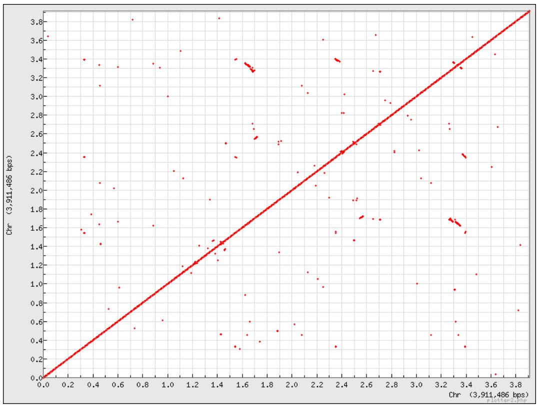

A

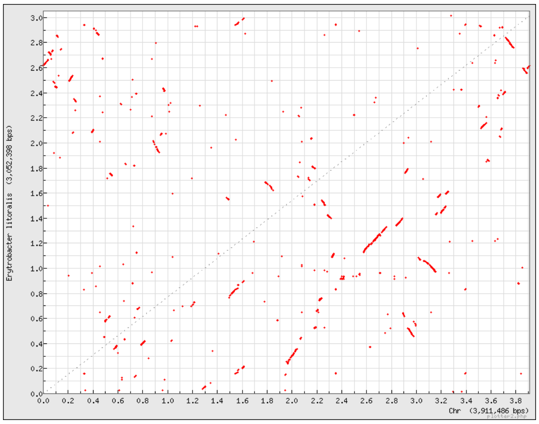

B

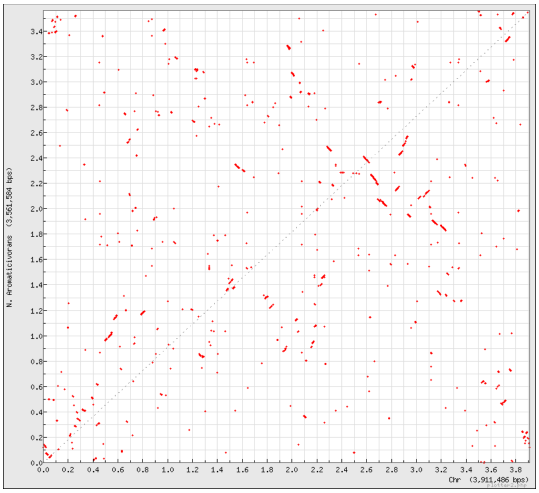

C

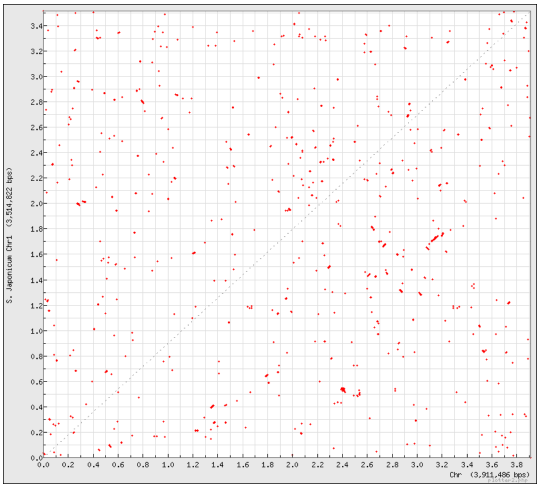

D

FIG S1 Dotplots of PP1Y Chr. The Chr proteome was compared with itself (A), and with those of *Erythrobacter litoralis* (B), *Novosphingobium aromaticivorans DSM 12444* (C), and *Sphingonium japonicum* (D).

A

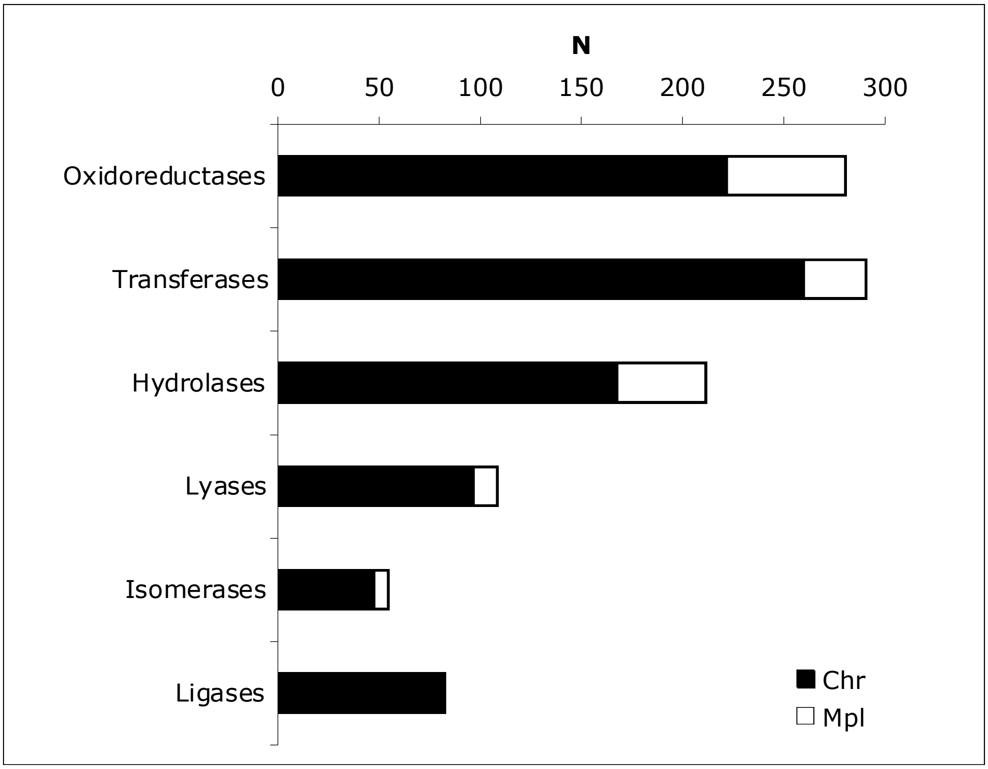

B

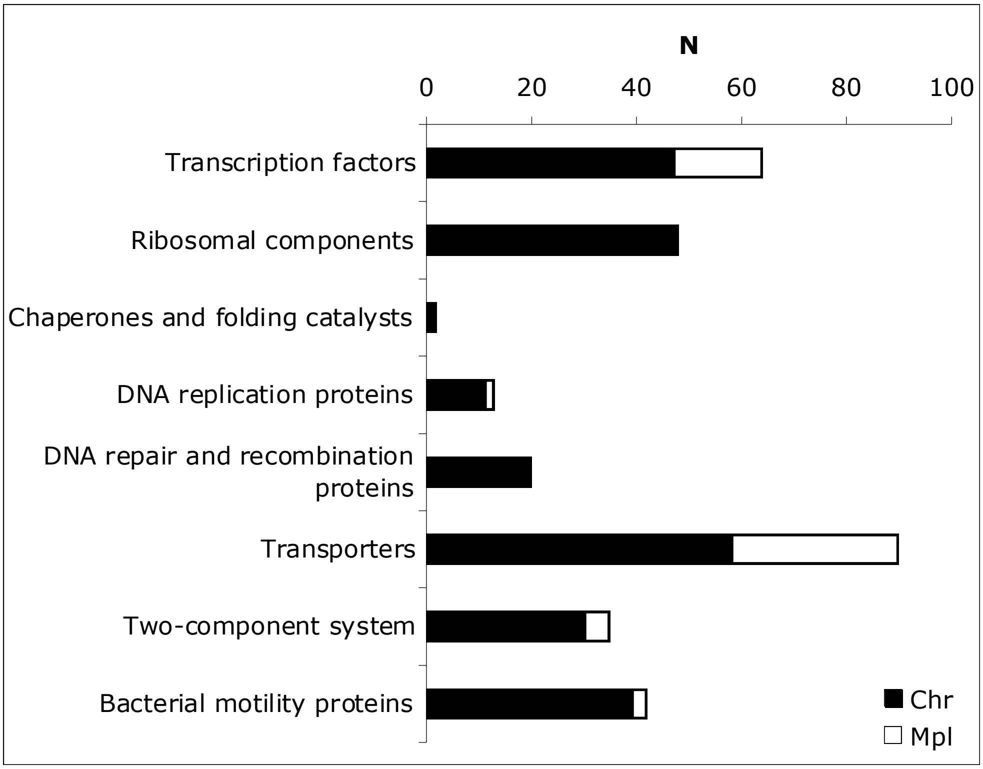

FIG S2 Genes and proteins. Distribution of Chr and Mpl genes with respect to enzyme classes (A) and most frequently found protein families according to KAAS (B). N, number of genes.

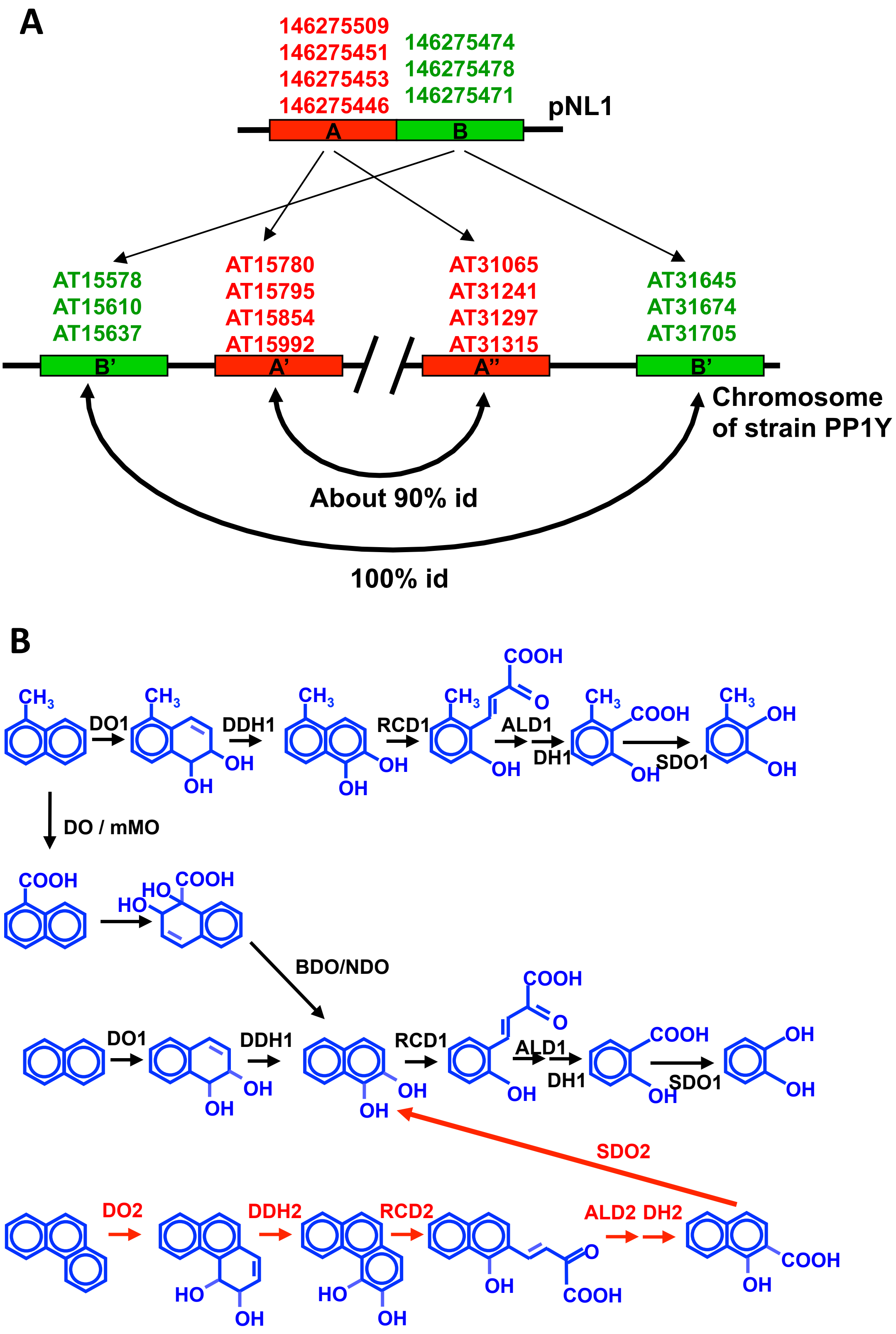

FIG S3 A) Regions of plasmid pNL1 duplicated on the chromosome of strain PP1Y. The ORFs coding for the alpha subunits of the dioxygenases contained in the regions A, A', A'', and B, B' are indicated above each box (gi accession numbers are shown in the case of pNL1). The 3 couples of ORFs sharing 100% identity are in the duplicate region B' whereas the 4 couples of ORFs sharing a 90-95% identity are in regions A' and A''. (B) Proposed pathway for the degradation of (methyl)naphthalene coded by plasmid pNL1 of strain F199 (black arrows). The pathway with the red arrows is a hypothetical expansion of the naphthalene pathway obtained by duplication and divergence of the ORFs involved in the metabolism of (methyl)naphthalene. DO, soluble dioxygenase; DDH dihydrodiol dehydogenase; RCD, ring cleavage dioxygenase; ALD, aldolase; DH, dehydrogenase; SDO, salicylate dioxygenase; mMO, membrane bound monooxygenase; BDO/NDO, benzoate/naphthoate dioxygenase.

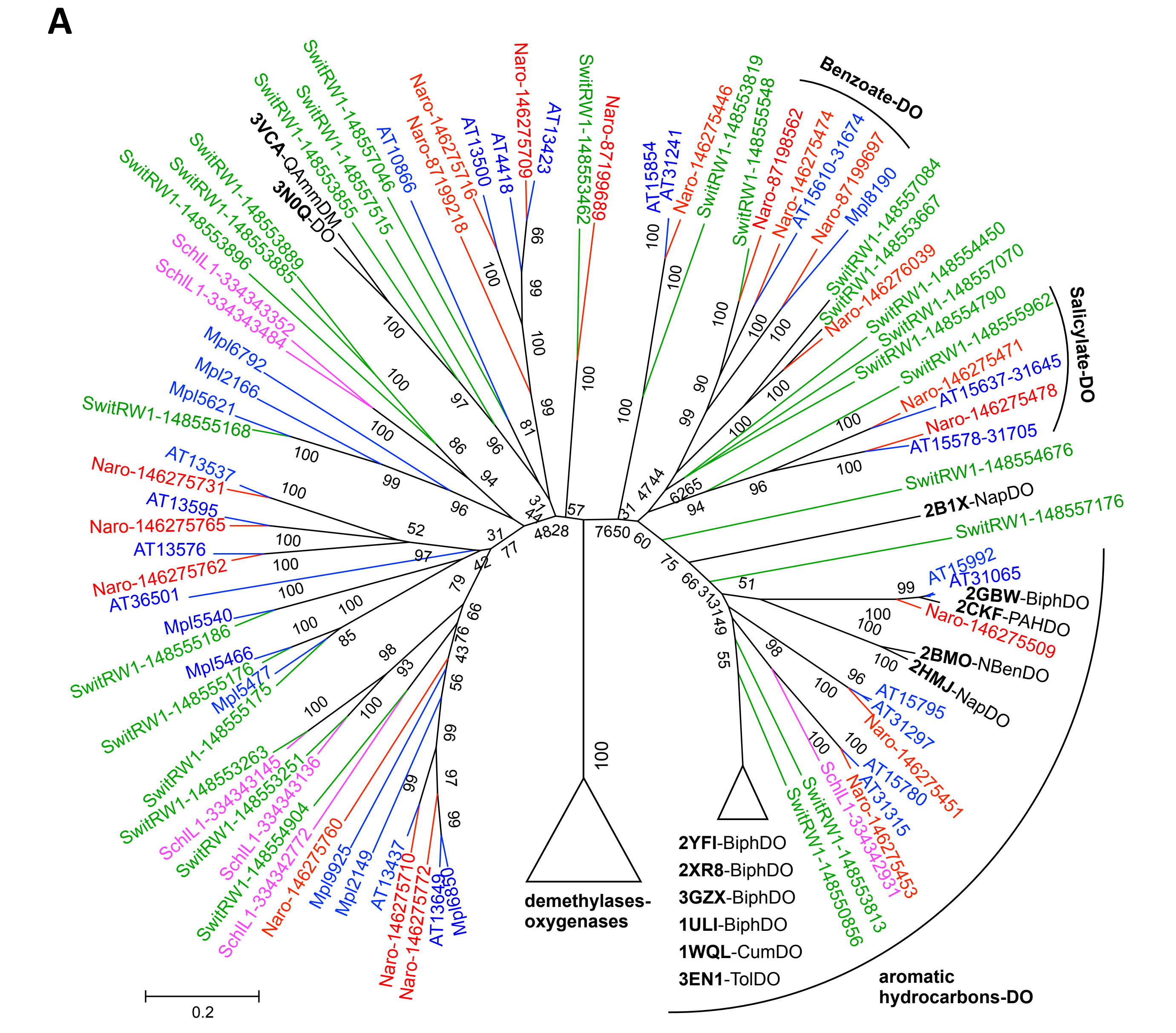

FIG S4 Neighbor-Joining tree summarizing the relationships between the alpha subunits of the oxygenases of strains PP1Y (blue), F199 (red), RW1 (green) and L-1 (magenta) and some dioxygenases whose structure has been determined (black). The analysis involved 146 amino acid sequences. For the sake of clarity the two divergent branches of the tree are shown separately in panels A and B. The genome of strain L-1 codes for only 6 oxygenases all in the branch of panel A. The genome of strain RW1 codes for 26 potential dioxygenases (panel A) and 32 potential oxygenases/demethylase (panel B). The numbers in the names of the oxygenases from *sphingomonadales* refer to the *gi* accession numbers of the NCBI protein database. PDB codes are in bold. BiphDO, biphenyl-DO; CumDO, cumene-DO; TolDO, toluene-DO; NBenDO, nitrobenzene-DO; NapDO, naphthaleneDO; PAHDO, PAH-DO; QAmmDM, quaternary ammonium-demethylase; CarDO, carbazole-DO; OQ8MO, 2-Oxoquinoline 8-Monooxygenase; 3Kst9Hy, 3-ketosteroid-9-alpha-hydroxylase, DicambaDM, Dicamba (3,6-dichloro-2-methoxybenzoic acid) demethylase.

B

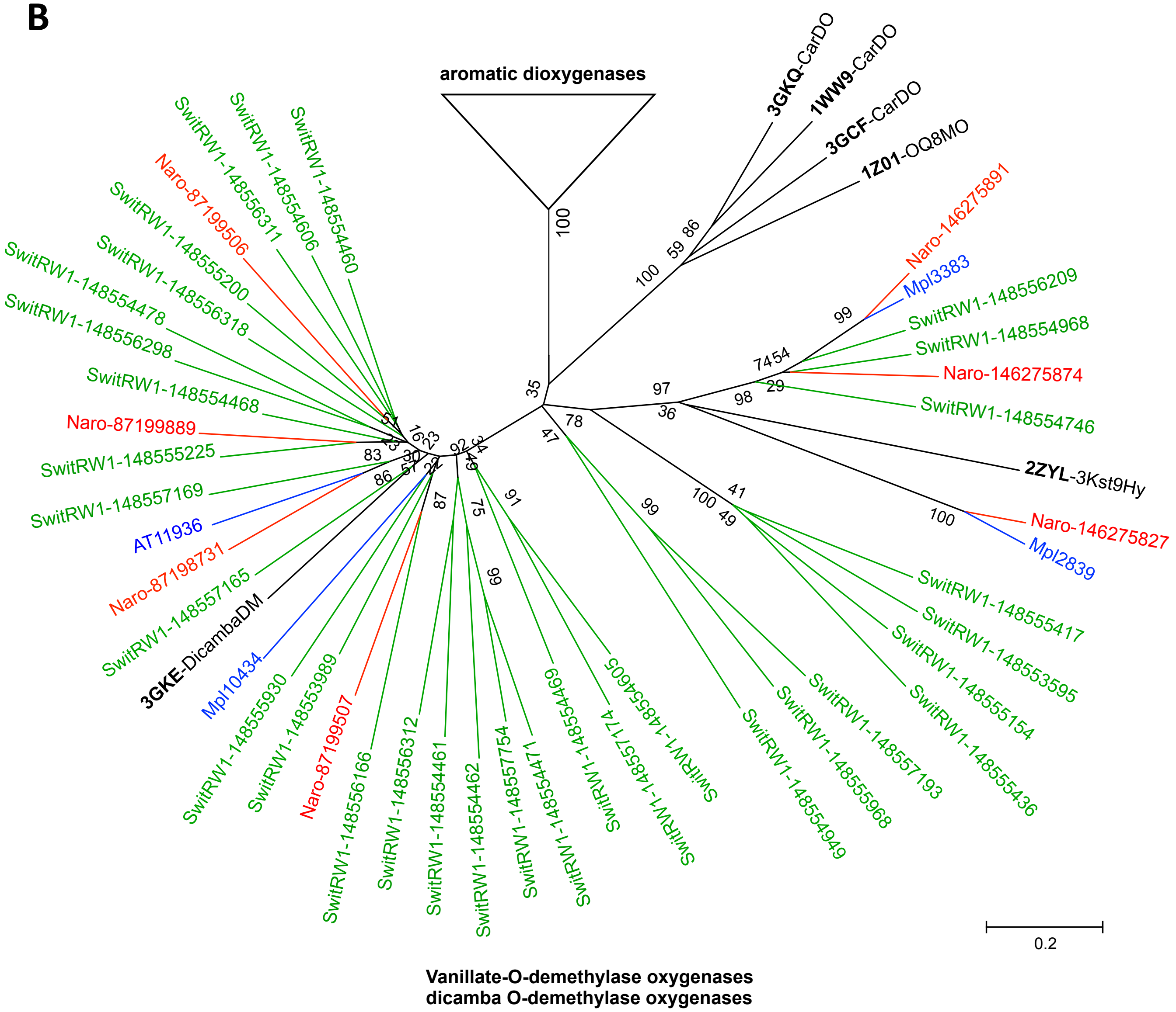

FIG S4 Neighbor-Joining tree summarizing the relationships between the alpha subunits of the oxygenases of strains PP1Y (blue), F199 (red), RW1 (green) and L-1 (magenta) and some dioxygenases whose structure has been determined (black). The analysis involved 146 amino acid sequences. For the sake of clarity the two divergent branches of the tree are shown separately in panels A and B. The genome of strain L-1 codes for only 6 oxygenases all in the branch of panel A. The genome of strain RW1 codes for 26 potential dioxygenases (panel A) and 32 potential oxygenases/demethylase (panel B). The numbers in the names of the oxygenases from *sphingomonadales* refer to the *gi* accession numbers of the NCBI protein database. PDB codes are in bold. BiphDO, biphenyl-DO; CumDO, cumene-DO; TolDO, toluene-DO; NBenDO, nitrobenzene-DO; NapDO, naphthaleneDO; PAHDO, PAH-DO; QAmmDM, quaternary ammonium-demethylase; CarDO, carbazole-DO; OQ8MO, 2-Oxoquinoline 8-Monooxygenase; 3Kst9Hy, 3-ketosteroid-9-alpha-hydroxylase, DicambaDM, Dicamba (3,6-dichloro-2-methoxybenzoic acid) demethylase.

```
PP38178,        1 MDALRYFLVPAMTLTGVAGFILGGPFVWLGIATFAVLMLLDIVLPSDHKARSRGIALVAD
PP37257,        1 MDALRYFLVPAMTLTGVAGFILGGPFVWLGIATFAVLMLLDIVLPSDHKVRSRGIAPVAD
                  ************************************************* ****** ***

PP38178,       61 IAIYLQFPLMVALYLAFANSVATGTNPIWGTDGSAWQLIGSIASLAWLSAVPTLPVAHEL
PP37257,       61 IAIYLQLPLIVALYLAFANSVVSGTNPIWGADGSAWQLIGSIASLAWLSAVPTLPVAHEL
                  ****** ** ***********  ******* *****************************

PP38178,      121 MHRRHWFPRAVAKGLSAFYGDPNRDVAHIVTHHVHLDTAKDSDTPLRGQTIYSFVFQATW
PP37257,      121 MHRRHWFPRAVAKGLSAFYGDPNRDVAHIVTHHVHLDTAKDSDTPLRGQTIYSFVFQATW
                  ************************************************************

PP38178,      181 GSYKDTWEKQGEILSRLGHSPWSWRNAMWLQLVLVGAIILGVAAAAGPIAGFATVGAMFF
PP37257,      181 GSYKDTWEKQGEILTRLGYSPWSWRNAMWLQLVLVGAIILGVAAAAGPIAGFATFGAMFF
                  ************** *** *********************************** *****

PP38178,      241 AKMFVEGFNYFQHYGLLRVEGDPIAKHHAWNHLGMIVRPIGVEITNHINHHLDGHIPFYE
PP37257,      241 AKMFVEGFNYFQHYGLLRVEGDPIAKHHAWNHLGMIVRPIGVEITNHINHHLDGHIPFYE
                  ************************************************************

PP38178,      301 LQPEPKAPQMPSLFLCFLCGLVPPVWHRFIAQPRLKDWDLHFASPSERKLAMAANAQAGW
PP37257,      301 LQPEPKAPQMPSLFLCFLCGLVPPVWHKFIAQPRLKDWDLHFASSTERQLAMAANARAGW
                  *************************** ****************  ** ******* ***

PP38178,      361 PAWATTD
PP37257,      361 PAWATTD
                  *******
```

FIG S5 Pairwise alignment between the two potential membrane monooxygenases of strain PP1Y. The differences are shown in red, hypothetical transmembrane helices in green, catalytic residues in blue. The red line indicates the hypothetical substrate binding region.

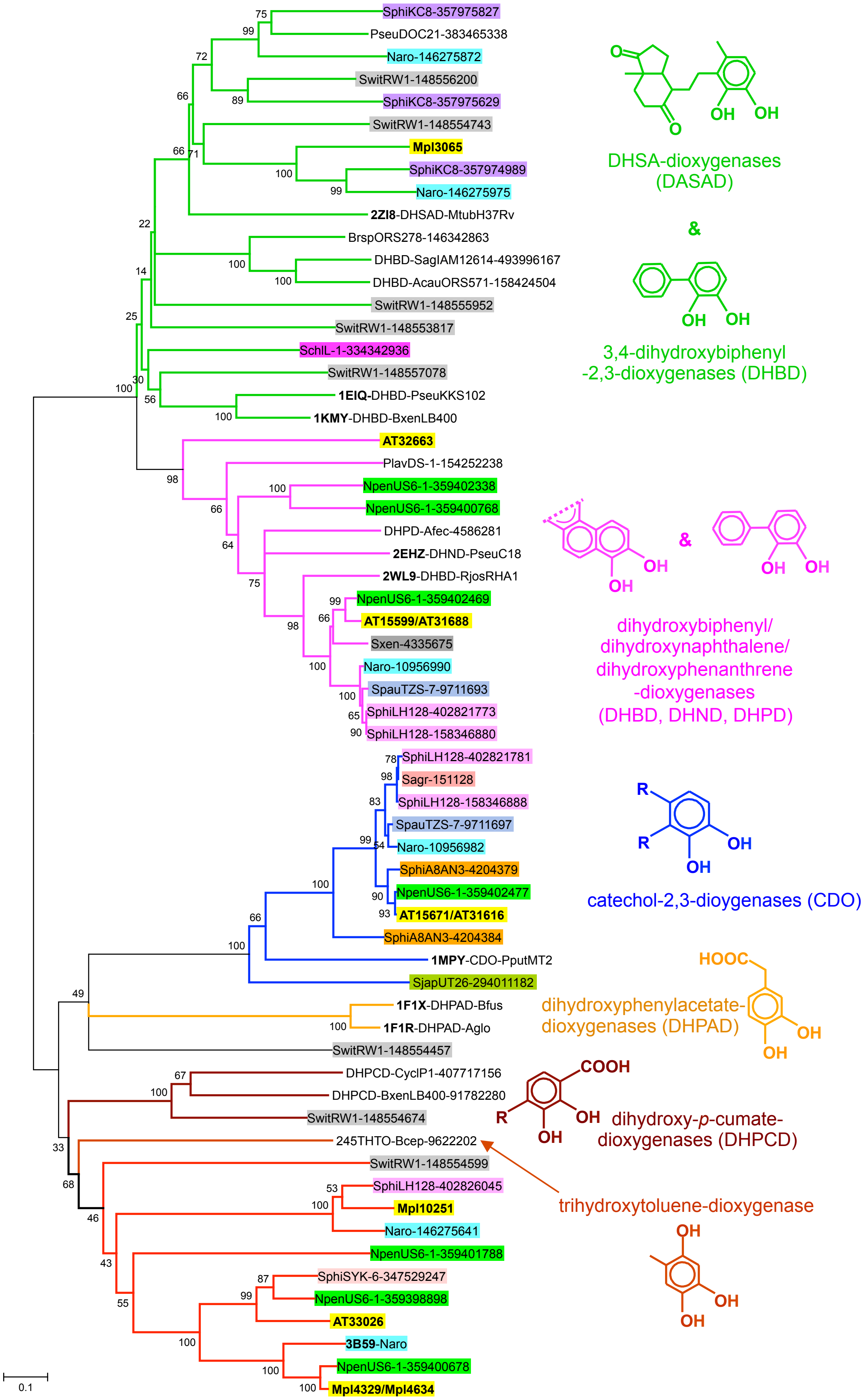

SphiKC8-357975827
PseuDOC21-383465338
Naro-146275872
SwitRW1-148556200
SphiKC8-357975629
SwitRW1-148554743
Mpl3065
SphiKC8-357974989
Naro-146275975
2ZI8-DHSAD-MtubH37Rv
BrspORS278-146342863
DHBD-SagIAM12614-493996167
DHBD-AcauORS571-158424504
SwitRW1-148555952
SwitRW1-148553817
SchlL-1-334342936
SwitRW1-148557078
1EIQ-DHBD-PseuKKS102
1KMY-DHBD-BxenLB400
AT32663
PlavDS-1-154252238
NpenUS6-1-359402338
NpenUS6-1-359400768
DHPD-Afec-4586281
2EHZ-DHND-PseuC18
2WL9-DHBD-RjosRHA1
NpenUS6-1-359402469
AT15599/AT31688
Sxen-4335675
Naro-10956990
SpauTZS-7-9711693
SphiLH128-402821773
SphiLH128-158346880
SphiLH128-402821781
Sagr-151128
SphiLH128-158346888
SpauTZS-7-9711697
Naro-10956982
SphiA8AN3-4204379
NpenUS6-1-359402477
AT15671/AT31616
SphiA8AN3-4204384
1MPY-CDO-PputMT2
SjapUT26-294011182
1F1X-DHPAD-Bfus
1F1R-DHPAD-Aglo
SwitRW1-148554457
DHPCD-CyclP1-407717156
DHPCD-BxenLB400-91782280
SwitRW1-148554674
245THTO-Bcep-9622202
SwitRW1-148554599
SphiLH128-402826045
Mpl10251
Naro-146275641
NpenUS6-1-359401788
SphiSYK-6-347529247
NpenUS6-1-359398898
AT33026
3B59-Naro
NpenUS6-1-359400678
Mpl4329/Mpl4634
0.1
DHSA-dioxygenases
(DASAD)
&
3,4-dihydroxybiphenyl
-2,3-dioxygenases (DHBD)
&
dihydroxybiphenyl/
dihydroxynaphthalene/
dihydroxyphenanthrene
-dioxygenases
(DHBD, DHND, DHPD)
catechol-2,3-dioygenases (CDO)
HOOC
dihydroxyphenylacetate-
dioxygenases (DHPAD)
COOH
dihydroxy-p-cumate-
dioxygenases (DHPCD)
trihydroxytoluene-dioxygenase

FIG S6. Neighbor-Joining tree summarizing the relationships among PP1Y RCDs (highlighted in yellow), the RCDs from several sphingomonads (each highlighted in a different color) and several other RCDs. The numbers in the names of the oxygenases refer to the *gi* accession numbers of the NCBI protein database. The PDB codes of known structures are shown in bold. Three of the PP1Y ORFs are present in double copy with a 100% identity. The AT15671/ AT31616 and AT15599/AT31688 couples are located on chromosome in the pNL1 derived duplicated region B' (Supplementary Figure S3), whereas the Mpl4329/Mpl4634 couple derives from a further duplication on the megaplasmid. The analysis involved 62 amino acid sequences. All positions containing gaps were eliminated. There were a total of 189 positions in the final dataset.

Abbreviations: Naro, *N. aromaticivorans* F199; SwitRW1, *S. wittichii* RW1; NpenUS6-1, *N. pentaromativorans* US6-1; SchlL1, *S. chlorophenolicum* L-1; SphiKC8, *Sphingomonas* sp. KC8; SphiSYK6, *Sphingobium* sp. SYK-6; SpauTZS7 *Sphingomonas paucimobilis* TZS-7; SphiLH128, *Sphingomonas* sp. LH128; SphiA8AN3, *Sphingomonas* sp. A8AN3; Sagr, *Sphingomonas agrestis*; Sxen, *Sphingobium xenophagum*; SagIAM12614, *Stappia aggregata* IAM 12614; PseuDOC21, *Pseudomonas* sp. DOC21; CyclP1, *Cycloclasticus* sp. P1; BxenLB400, *Burkholderia xenovorans* LB400; MtubH37Rv, *Mycobacterium tuberculosis* H37Rv; PseuC18, *Pseudomonas* sp. C18; PputMT2, *Pseudomonas* putida MT2; Bfus, *Brevibacterium fuscum*; Aglo, *Arthrobacter globiformis*; RjosRHA1, *Rhodococcus jostii* RHA1; Afec, *Alcaligenes faecalis*; Bcep, *Burkholderia cepacia*; PseuKKS102, Pseudomonas sp. KKS102; BrspORS278, *Bradyrhizobium* sp. ORS278; AcauORS571, *Azorhizobium caulinodans* ORS 571; PlavDS-1, *Parvibaculum lavamentivorans* DS-1.

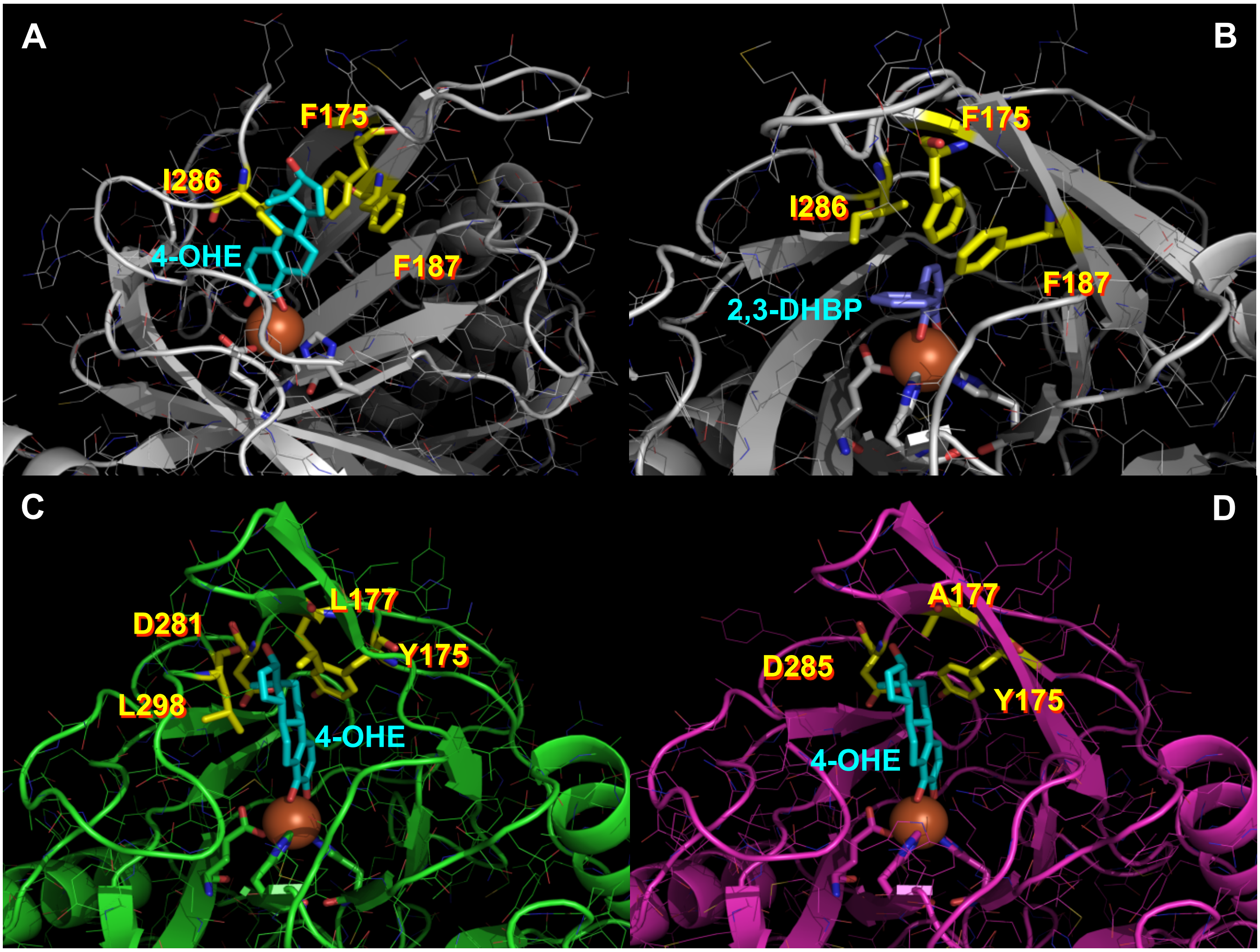

FIG S7 Homology models of PP1Y RCDs. A and B show models of the RCD coded by Mpl3065 with 4-hydroxyoestradiol (4-OHE) and 2,3-dihydroxybiphenyl (2,3-DHBP), respectively, docked into the active site. The active site of this enzyme can host only catechols with substituents at position 3 which can adopt an orientation perpendicular to the catechol ring, like 2,3-dihydroxybiphenyl, propylcatechol and isopropylcatechol. This is due to the steric hindrance of residues F175, F187 and I286 that prevent the positioning of polycyclic catechols. C and D show models of the RCDs coded by AT15599/AT31688 and AT32663, respectively, with 4-OHE docked into the active site. The active site pockets of both RCDs are wider than that of Mpl3065 and opened to the solvent. In particular, AT32663, due to a deletion of the C-terminus, has a very wide active site. Both enzymes should be able to bind substrates with 3-4 rings or larger.

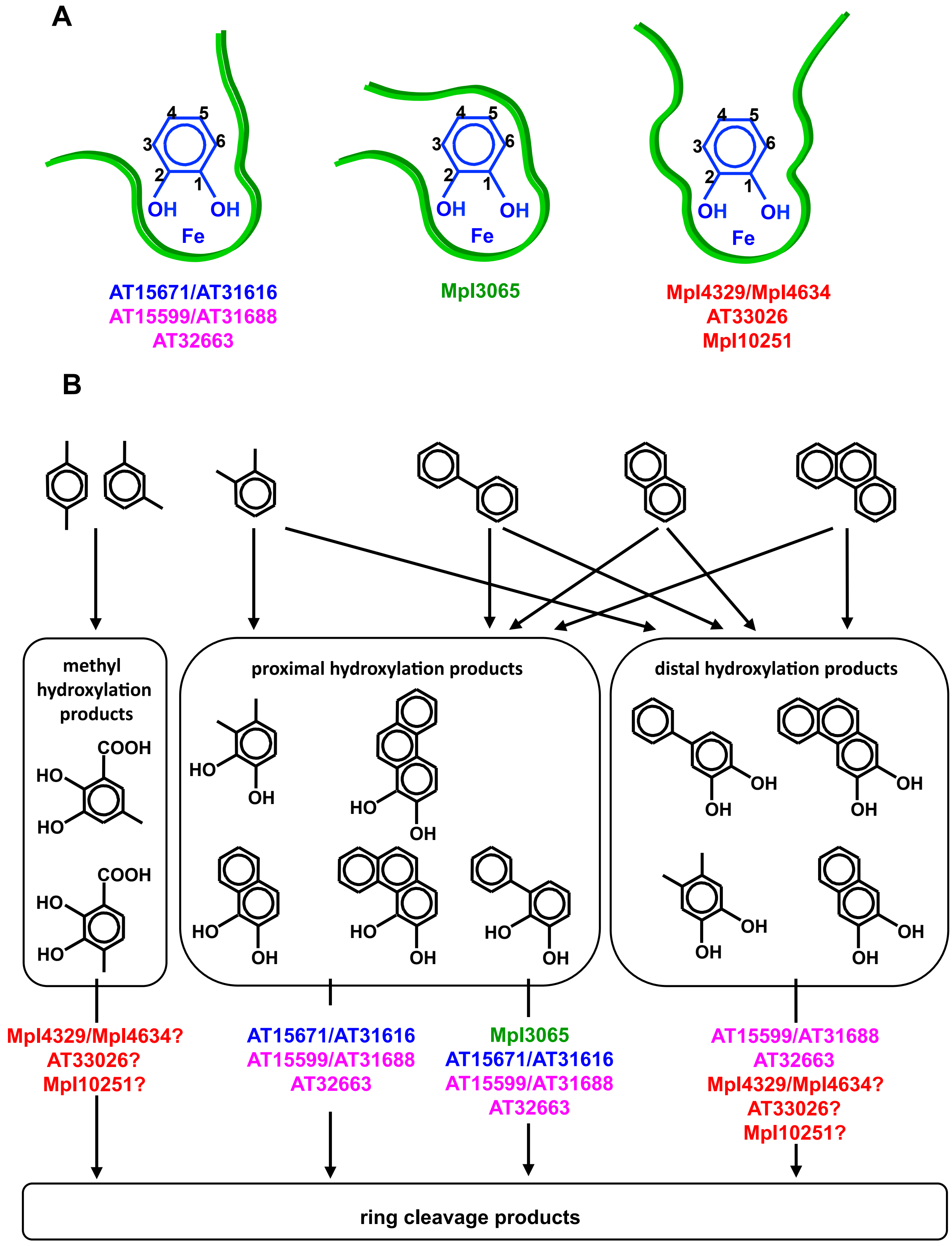

FIG S8 A) Schematic drawing of the active sites of the 7 RCDs from strain PP1Y. B) Possible metabolism of mono and polycyclic aromatic hydrocarbons during growth on complex mixtures of hydrocarbons (e.g. gasoline and diesel oil).

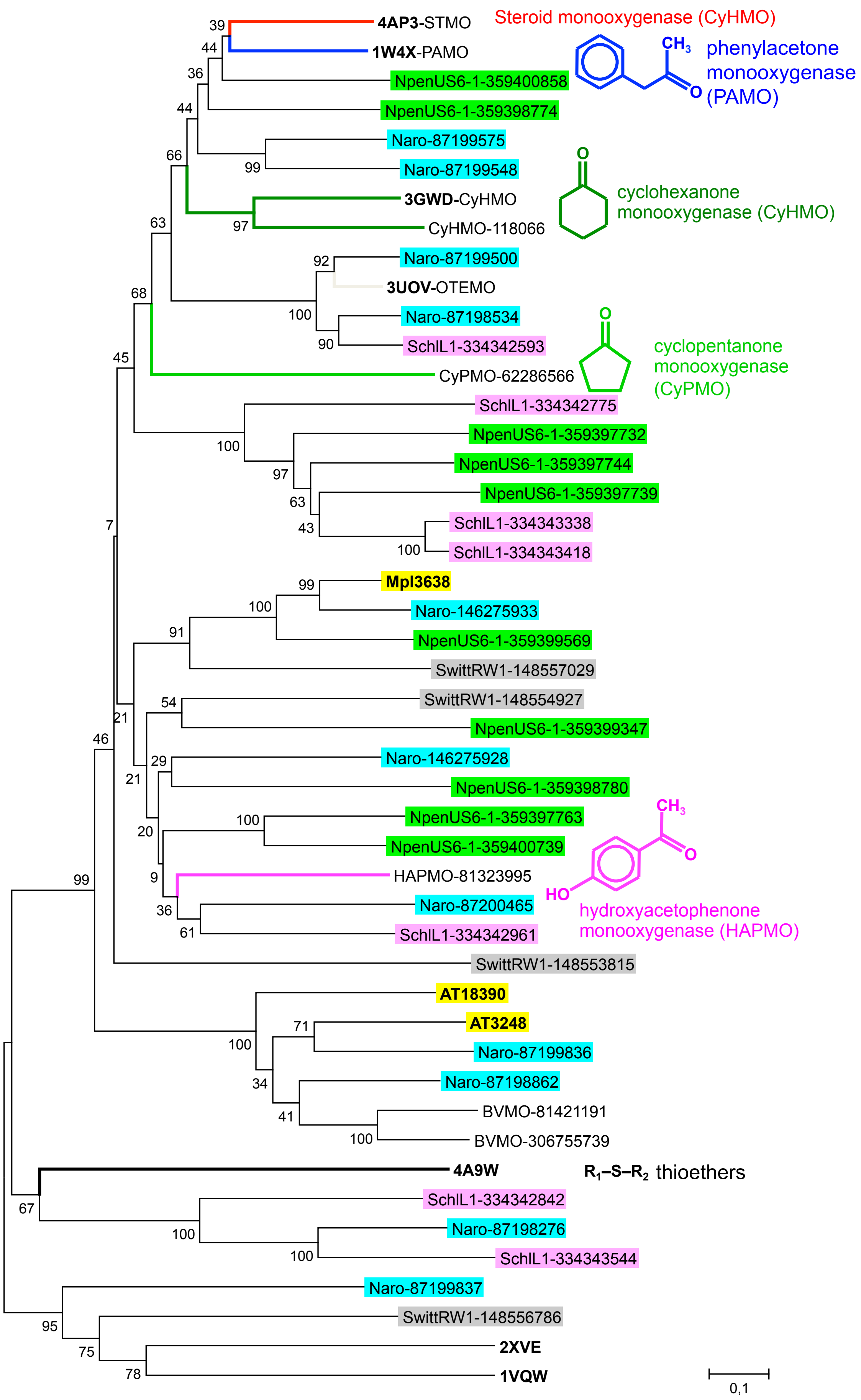
4AP3-STMO
Steroid monooxygenase (CyHMO)
1W4X-PAMO
phenylacetone monooxygenase (PAMO)
NpenUS6-1-359400858
NpenUS6-1-359398774
Naro-87199575
Naro-87199548
3GWD-CyHMO
CyHMO-118066
cyclohexanone monooxygenase (CyHMO)
Naro-87199500
3UOV-OTEMO
Naro-87198534
SchlL1-334342593
CyPMO-62286566
cyclopentanone monooxygenase (CyPMO)
SchlL1-334342775
NpenUS6-1-359397732
NpenUS6-1-359397744
NpenUS6-1-359397739
SchlL1-334343338
SchlL1-334343418
Mpl3638
Naro-146275933
NpenUS6-1-359399569
SwittRW1-148557029
SwittRW1-148554927
NpenUS6-1-359399347
Naro-146275928
NpenUS6-1-359398780
NpenUS6-1-359397763
NpenUS6-1-359400739
HAPMO-81323995
Naro-87200465
SchlL1-334342961
hydroxyacetophenone monooxygenase (HAPMO)
SwittRW1-148553815
AT18390
AT3248
Naro-87199836
Naro-87198862
BVMO-81421191
BVMO-306755739
4A9W
R_1–S–R_2 thioethers
SchlL1-334342842
Naro-87198276
SchlL1-334343544
Naro-87199837
SwittRW1-148556786
2XVE
1VQW
0,1

FIG S9 Neighbor-Joining tree summarizing the relationships among PP1Y BVMOs (highlighted in yellow), the BVMOs from several sphingomonads (each highlighted in a different color) and several other BVMOs. The numbers in the name of the oxygenases refer to the *gi* accession numbers of the NCBI protein database. PDB codes of known structures are shown in bold. The analysis involved 47 amino acid sequences. All positions containing gaps were eliminated. There were a total of 222 positions in the final dataset. OTEMO is 2-oxo-Δ(3)-4,5,5-trimethylcyclopentenylacetyl-CoA monooxygenase, which catalyses a key step in the metabolism of camphor by *Pseudomonas putida* ATCC 17453.

Abbreviations as in Supplementary Figure S6.

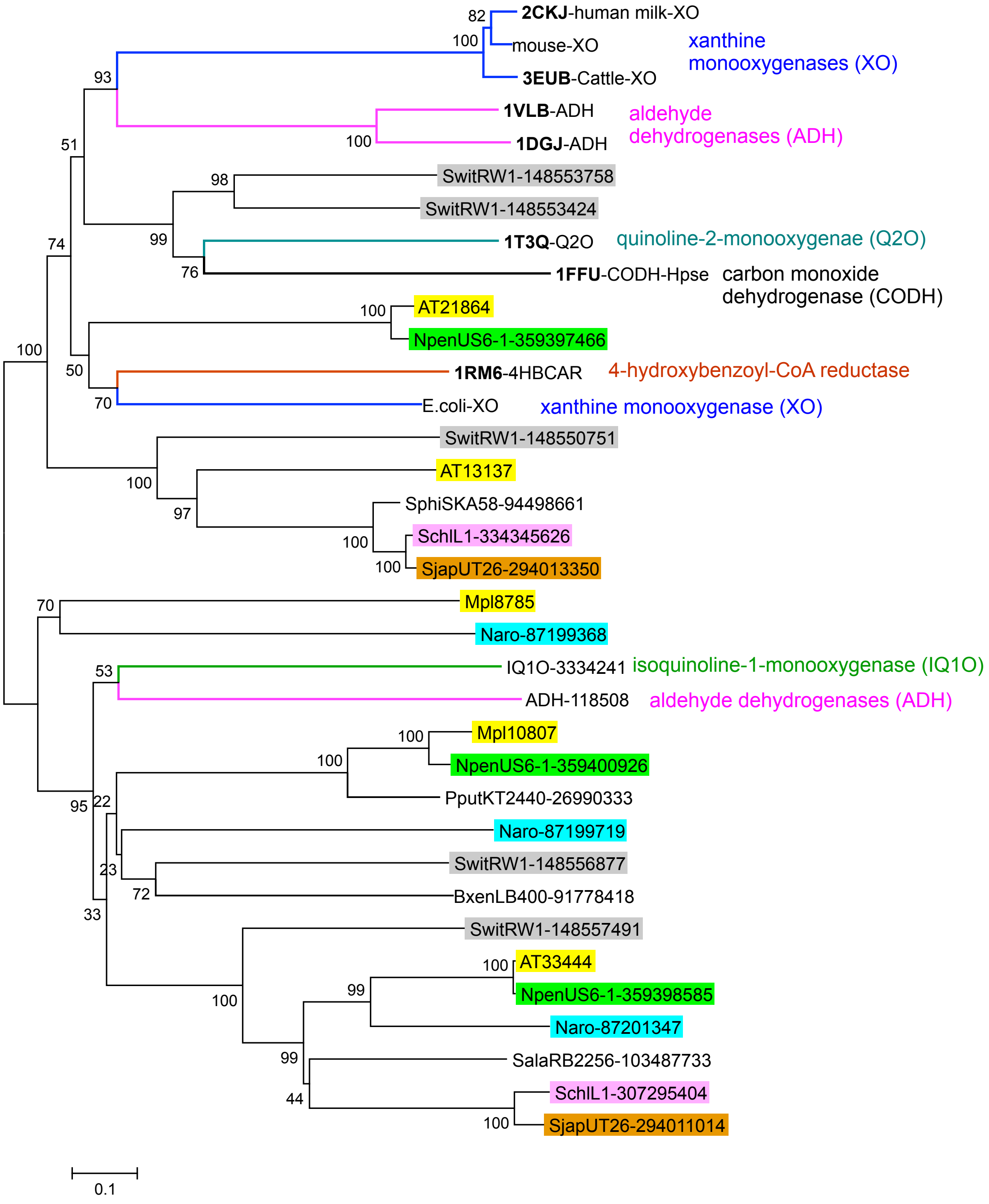

FIG S10 Neighbor-Joining tree summarizing the relationships among PP1Y MoMOs (highlighted in yellow), the MoMOs from several sphingomonads (each highlighted in a different color) and several other BVMOs. The numbers in the name of the oxygenases refer to the *gi* accession numbers of the NCBI protein database. PDB codes of known structures are shown in bold. The analysis involved 35 amino acid sequences. All positions containing gaps were eliminated. There was a total of 374 positions in the final dataset. Abbreviations: SphiSKA58, *Sphinogomonas* sp; SKA58; SalaRB2256, *Sphingopyrix alaskensis RB2256; PputKT2440, Pseudomonas putida KT2440.* Other abbreviations are as in Supplementary Figure S6.

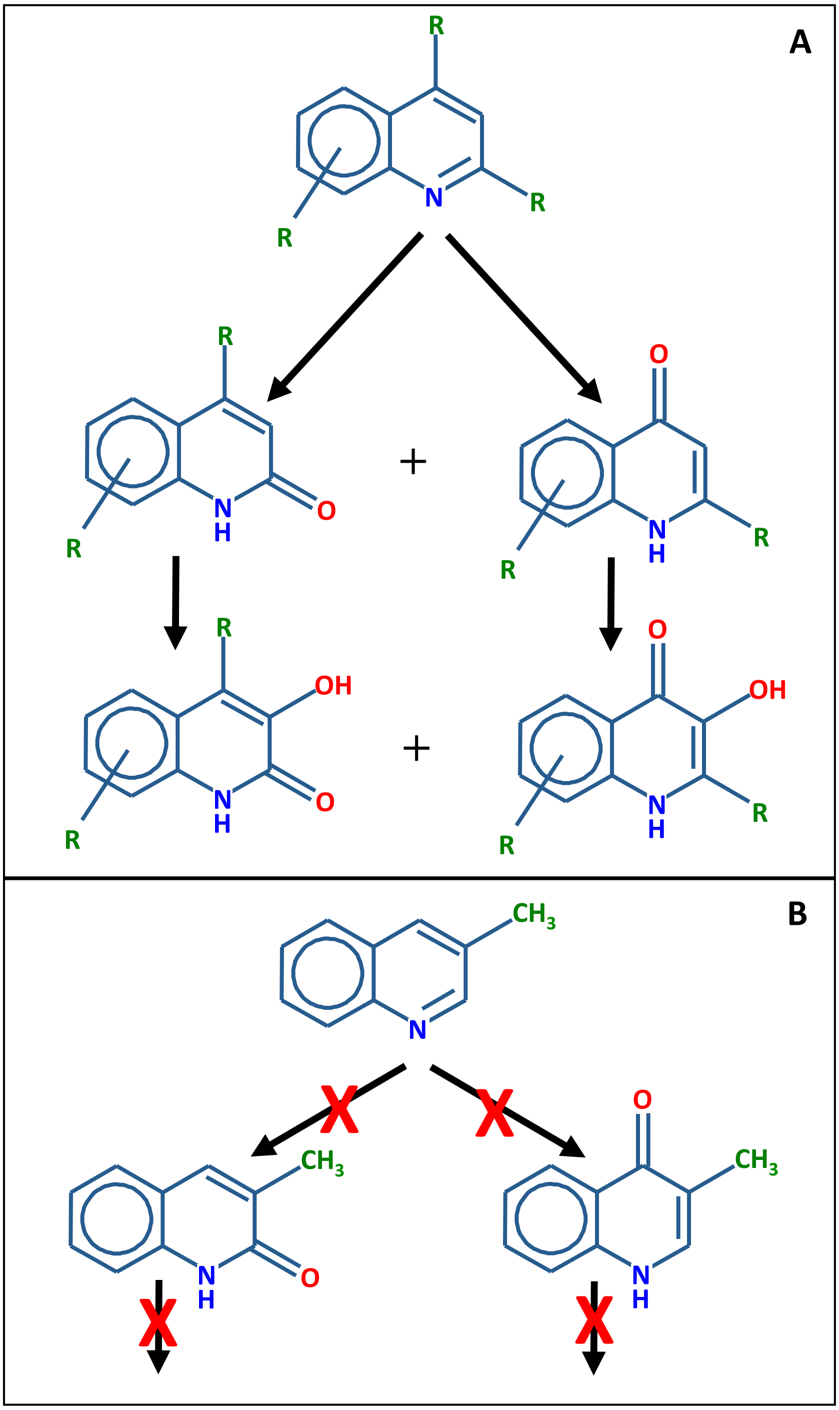

FIG S11 Hypothetical hydroxylation reactions involved in the degradation of methylquinolines. A) Initial hydroxylation at positions 2 and 4 followed by hydroxylation at position 3. Both pathways have been already described for quinoline degrading bacteria. B) A methyl group at position 3 (3-methylquinoline) could inhibit the first and or the second hydroxylation reaction, which explains why 3-methylquinoline is not a growth substrate.

A) RND type Efflux Pumps (Inner Membrane subunit)

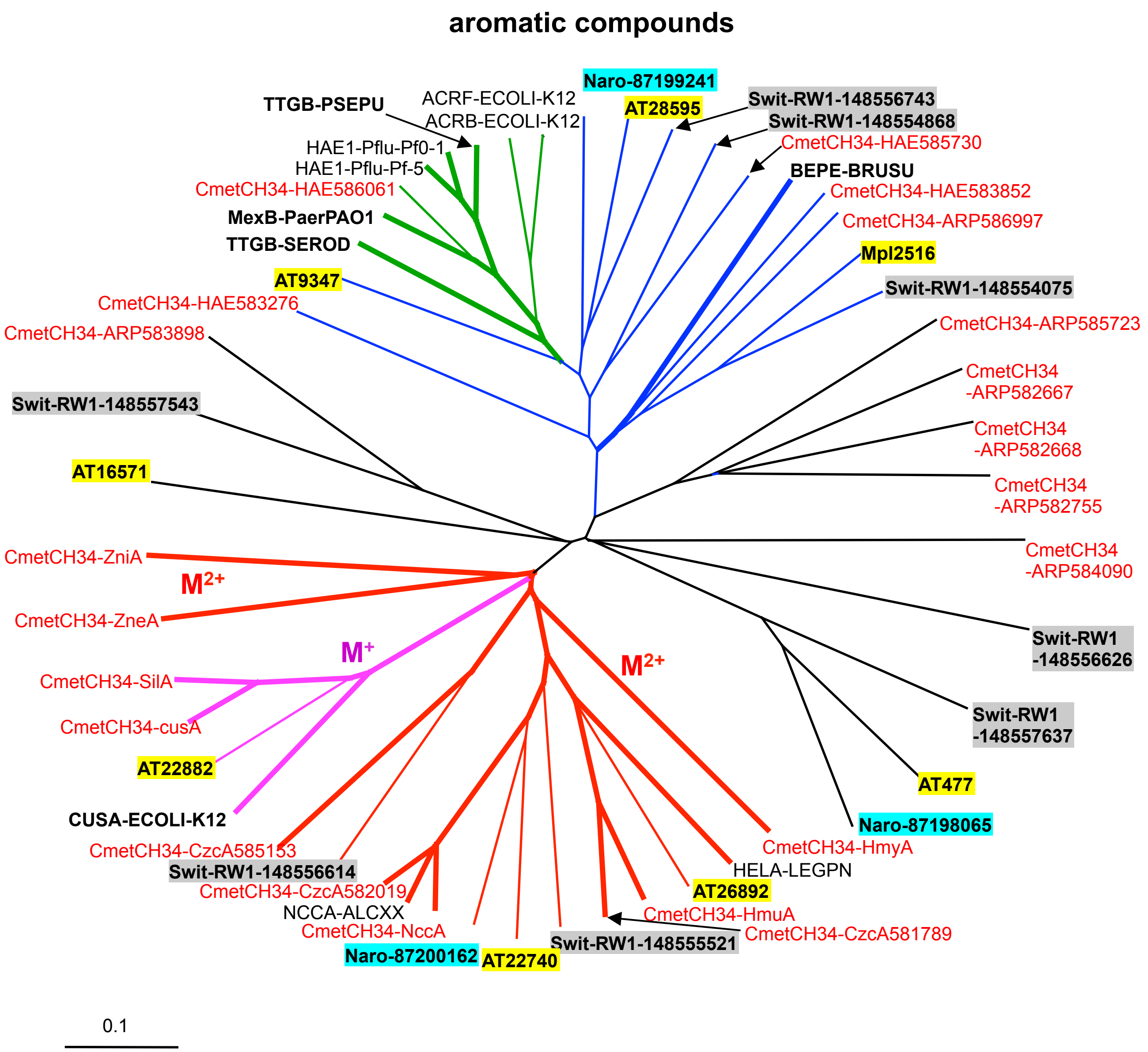

B) P-type-ATPases

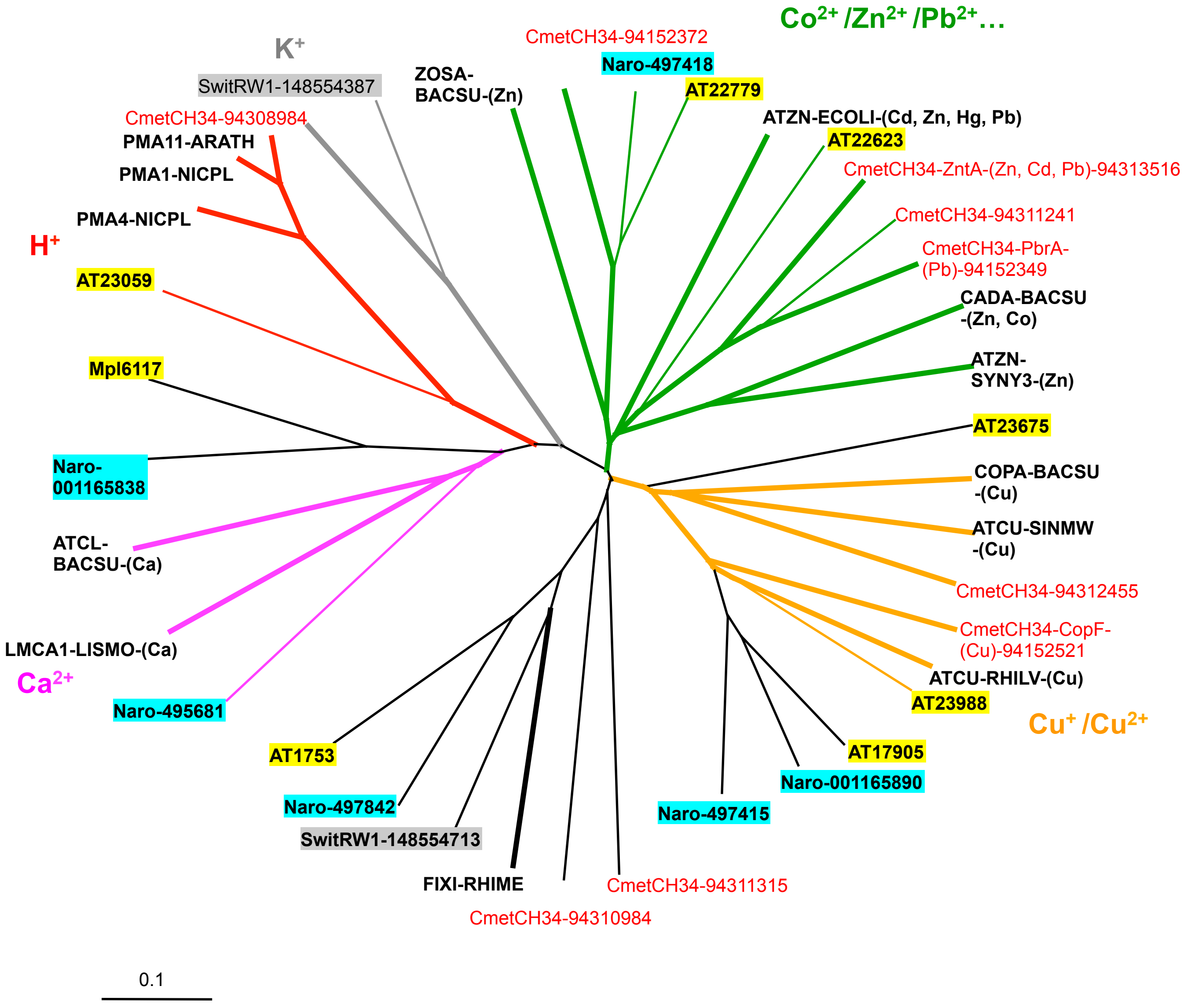

FIG S12 ClustalW tree showing the evolutionary relationships among the inner membrane subunit of RND-type efflux pumps (A) and the P-type ATPases (B). Proteins from Sphingomonads are highlighted. The numbers in the name of the oxygenases refer to the gi accession numbers of the NCBI protein database. The sequences shown in red came from *Cupriavidus metallidurans* CH34, a benchmark among the strains able to tolerate very high concentrations of transition metals.

In (A), the blue/green branch define a subfamily of RND pumps specific for neutral aromatic molecules like aromatic hydrocarbons (green branches) and acriflavine. The red/magenta branch define a subfamily of RND pumps specific for mono (magenta) and divalent (red) transition metals. None of the proteins belonging to the intermediate branches shown in black have been characterized, therefore it is not possible to speculate about the physiological role of the AT477 and AT16571 PP1Y RND-type pumps. Abbreviations: MexB-PaerPAO1, multidrug efflux transporter MexB from *Pseudomonas aeruginosa* PAO1; TTGB-PfluPf5, multidrug/solvent transporter TtgB from *Pseudomonas fluorescens* Pf-5; HAE1-PfluPf01, hydrophobe/amphiphile efflux-1 HAE1 from *P. fluorescens* Pf0-1; TTGB-Pput, toluene efflux pump TtgB from *Pseudomonas putida*; TTGB-Sodo, toluene efflux pump TtgB from *Serratia odorifera*; ACRF-EcolK12, acriflavine resistance protein F from *E. coli* K12; ACRB-EcolK12, acriflavine resistance protein B from *E. coli* K12; NCCA-Axyl, nickel-cobalt-cadmium resistance protein NccA from *Alcaligenes xylosoxydans*; CUSA-EcolK12, cation efflux system protein CusA from E. coli K12; BEPE-Bsui, efflux pump BepE from *Brucella suis. Cupriavidus metallidurans* CH34 (CmetCH34) proteins: HmuA, heavy metal cation tricomponent efflux pump; NccA, proton antiporter cation efflux protein; HmyA, heavy metal cation tricomponent efflux pump; ZneA, heavy metal cation tricomponent efflux pump; ZniA, heavy metal cation tricomponent efflux pump; CusA, copper and silver tricomponent efflux pump; SilA, proton antiporter cation efflux protein; CzcA, heavy metal efflux pump. Other abbreviation as in Supplementary Figure S6.

In (B), the orange branches define a subfamily of P-type ATPases specific for copper, the green branches a subfamily able to excrete the divalent toxic metals cobalt, nickel, lead, cadmium, mercury, and zinc, the gray branches a subfamily specific for potassium, the magenta branches a subfamily specific for calcium and the black branches uncharacterized subfamilies. The red branches define a subfamily of H^+ transporters found only in plants. The PP1Y P-type ATPase belonging to this subfamily (coded by AT23059) was probably acquired by horizontal gene transfer. It could generate an H^+ gradient at the expense of ATP hydrolysis thus providing energy to the H^+ dependent RND type pumps. When known, the specificity is reported in parentheses. Abbreviations: CADA-Bsub, cadmium, zinc and cobalt-transporting ATPase from *B. subtilis*; ZOSA-Bsub, zinc transporting ATPase from *B. subtilis*; COPA-Bsub, copper-exporting ATPase A from *B. subtilis*; ATCU-Smed, copper-transporting ATPase from Sinorhizobium medicae WSM419; ATZN-Syne, zinc-transporting ATPase from *Synechocystis* sp. ATCC 27184; ATCU-Rleg, copper-transporting ATPase from Rhizobium leguminosarum; ATZN-EcolK12, lead, cadmium, zinc and mercury-transporting ATPase from *E. coli* K-12; LMCA-Lmon calcium-transporting ATPase from *Listeria monocytogenes*; ATCL-Bsub, calcium-transporting ATPase from *B. subtilis*; PMA1-Nplu, plasma membrane ATPase 1 from *Nicotiana plumbaginifolia*; PMA4-Nplu, plasma membrane ATPase 4 from *N. plumbaginifolia*; PMA11-Atha, plasma membrane-type ATPase 11 from *Arabidopsis thaliana*; FIXI-Rmel, nitrogen fixation protein FixI from *Rhizobium meliloti*. *C. metallidurans* CH34 proteins: CopF, copper efflux ATPase; PbrA, Pb(II) resistance ATPase; ZntA, ATPase involved in Zn(II), Cd(II), Tl(I) and Pb(II) resistance; CzcP, cation efflux ATPase.

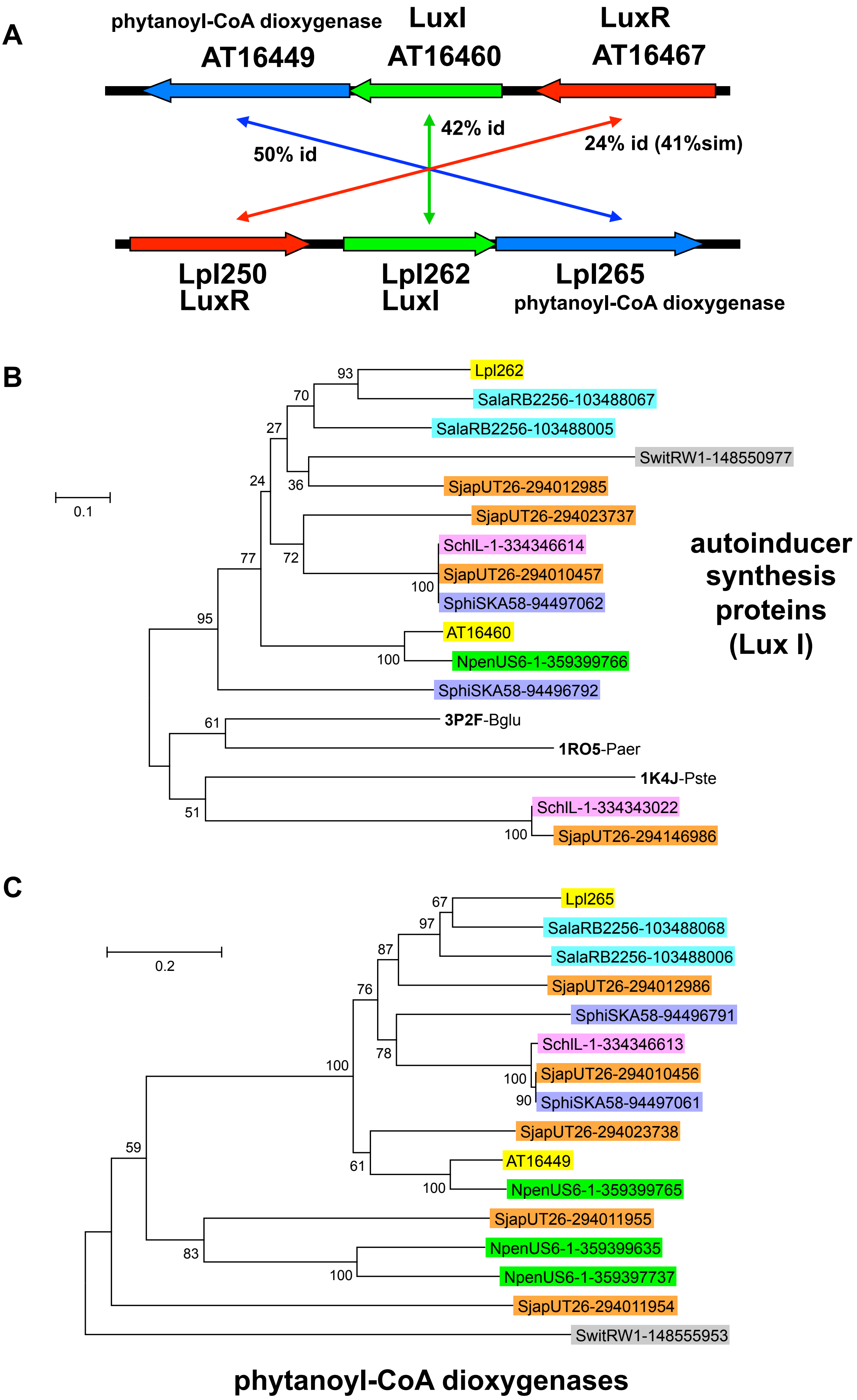

A
phytanoyl-CoA dioxygenase
AT16449
LuxI
AT16460
LuxR
AT16467
42% id
50% id
24% id (41%sim)
Lpl250
LuxR
Lpl262
LuxI
Lpl265
phytanoyl-CoA dioxygenase
B
0.1
Lpl262
SalaRB2256-103488067
SalaRB2256-103488005
SwitRW1-148550977
SjapUT26-294012985
SjapUT26-294023737
SchIL-1-334346614
SjapUT26-294010457
SphiSKA58-94497062
AT16460
NpenUS6-1-359399766
SphiSKA58-94496792
3P2F-Bglu
1RO5-Paer
1K4J-Pste
SchIL-1-334343022
SjapUT26-294146986
93
70
27
36
24
72
77
100
95
100
61
51
100
autoinducer synthesis proteins (Lux I)
C
0.2
Lpl265
SalaRB2256-103488068
SalaRB2256-103488006
SjapUT26-294012986
SphiSKA58-94496791
SchIL-1-334346613
SjapUT26-294010456
SphiSKA58-94497061
SjapUT26-294023738
AT16449
NpenUS6-1-359399765
SjapUT26-294011955
NpenUS6-1-359399635
NpenUS6-1-359397737
SjapUT26-294011954
SwitRW1-148555953
67
97
87
76
78
100
90
100
61
100
59
83
100
phytanoyl-CoA dioxygenases

FIG S13 Cluster of the ORFs coding for LuxR, LuxI and a phytanoyl-CoA dioxygenase like protein located on chromosome and on the large plasmid LpI (A). Neighbor-Joining tree showing the evolutionary relationships among LuxI (B) and phytanoyl-CoA dioxygenase (C) in different sphingomonads.

Abbreviation: SphiSKA58, *Sphinogomonas* sp SKA58; SalaRB2256, *Sphingopyxis alaskensis* RB2256; Bgluy,; *Burkholderia glumae;* Paer, *Pseudomonas aeruginosa;* Pste, *Pantoea stewartii.* Other abbreviations as in Supplementary Figure S6. PDB codes are shown in boldface type.

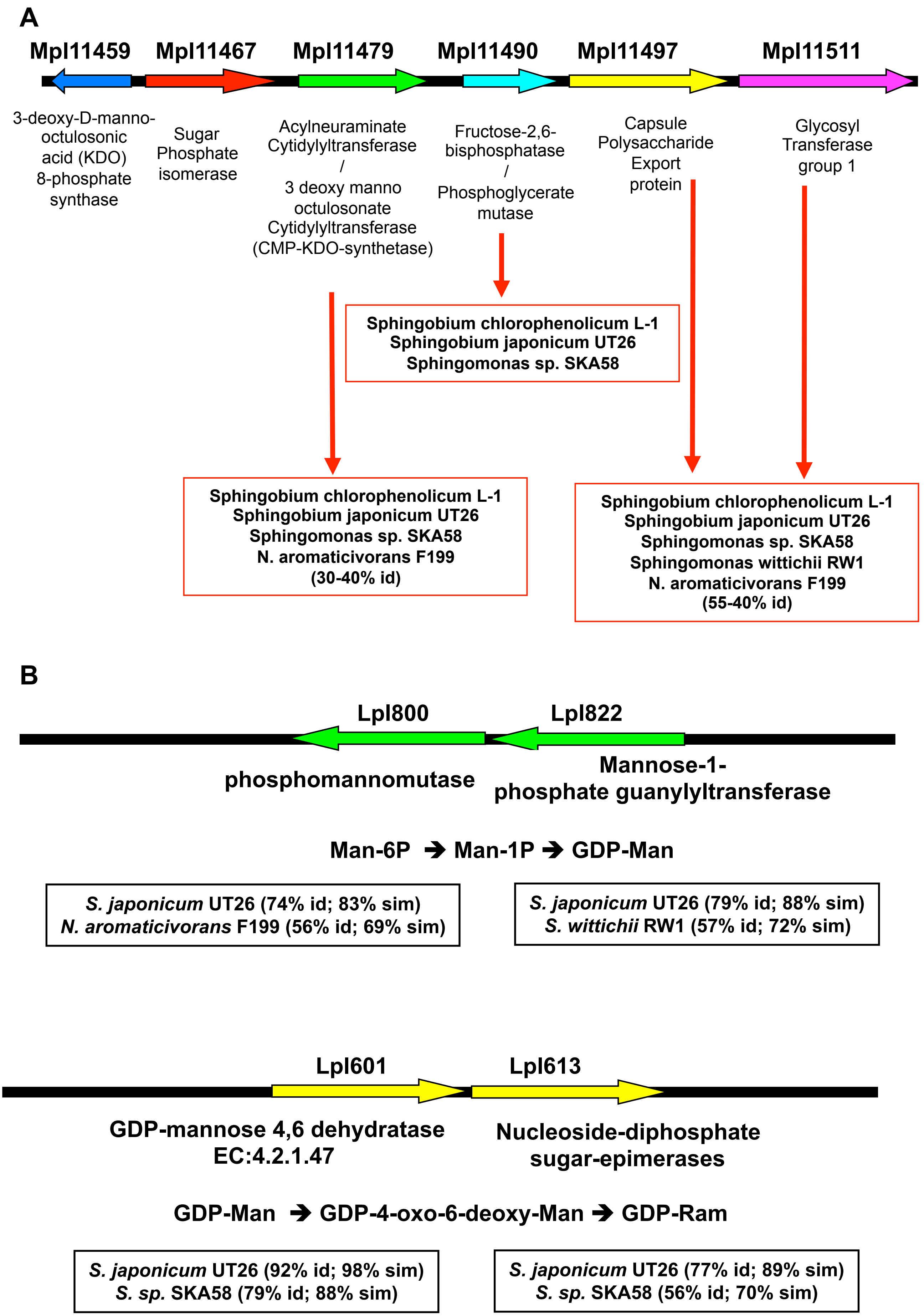
A
Mpl11459
Mpl11467
Mpl11479
Mpl11490
Mpl11497
Mpl11511
3-deoxy-D-manno-octulosonic acid (KDO) 8-phosphate synthase
Sugar Phosphate isomerase
Acylneuraminate Cytidylyltransferase / 3 deoxy manno octulosonate Cytidylyltransferase (CMP-KDO-synthetase)
Fructose-2,6-bisphosphatase / Phosphoglycerate mutase
Capsule Polysaccharide Export protein
Glycosyl Transferase group 1
Sphingobium chlorophenolicum L-1
Sphingobium japonicum UT26
Sphingomonas sp. SKA58
Sphingobium chlorophenolicum L-1
Sphingobium japonicum UT26
Sphingomonas sp. SKA58
N. aromaticivorans F199
(30-40% id)
Sphingobium chlorophenolicum L-1
Sphingobium japonicum UT26
Sphingomonas sp. SKA58
Sphingomonas wittichii RW1
N. aromaticivorans F199
(55-40% id)
B
Lpl800
Lpl822
phosphomannomutase
Mannose-1-phosphate guanylyltransferase
Man-6P ➔ Man-1P ➔ GDP-Man
S. japonicum UT26 (74% id; 83% sim)
N. aromaticivorans F199 (56% id; 69% sim)
S. japonicum UT26 (79% id; 88% sim)
S. wittichii RW1 (57% id; 72% sim)
Lpl601
Lpl613
GDP-mannose 4,6 dehydratase EC:4.2.1.47
Nucleoside-diphosphate sugar-epimerases
GDP-Man ➔ GDP-4-oxo-6-deoxy-Man ➔ GDP-Ram
S. japonicum UT26 (92% id; 98% sim)
S. sp. SKA58 (79% id; 88% sim)
S. japonicum UT26 (77% id; 89% sim)
S. sp. SKA58 (56% id; 70% sim)

FIG S14 Clusters of ORFs potentially coding for the synthesis of extracellular polysaccharides on the Mpl (A). Regions probably involved in the synthesis of exopolysaccharides on the Lpl (B). Two close couples of ORFs code for the enzymes necessary for the synthesis of the precursors GDP-mannose and GDP-rhamnose, i.e., the products of Lpl800 and Lpl822 convert mannose-6-phosphate to GDP-mannose, whereas the products of Lpl601 and Lpl613 convert GDP-mannose to GDP-rhamnose.

A

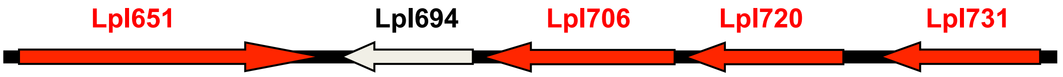

Lpl651 (1257aa): glycosyl transferase group 1
Lpl694: hypothetical protein
Lpl706 (395aa): glycosyl transferase
Lpl720 (394aa): glycosyl transferase
Lpl731 (420aa): putative glycosyl transferase

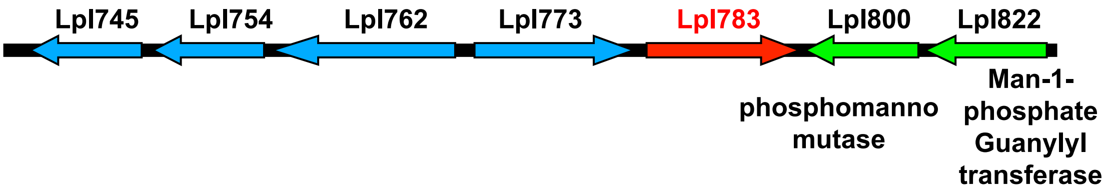

Lpl745 (223aa): ABC-type polysaccharide transport system, ATPase component
Lpl754 (265aa): ABC-
type polysaccharide export systems, permease component
Lpl762 (369aa): capsular polysaccharide transport system permease protein
Lpl773 (395aa): polysaccharide export outer membrane protein
Lpl783 (430aa): glycosyltransferase putative EC:2.4.1.

B

Lpl651: Not present in other sphingomonadas

Lpl706: Only in S. japonicum UT26 (29% id)

Lpl720: Not present in other sphingomonadas

Lpl731: Not present in other sphingomonadas

Lpl745: *N. aromaticivorans* F199 (69% id; 86% sim); S. sp. SKA58 (55% id; 73% sim)

Lpl754: *S. japonicum* UT26 (83% id; 92% sim); *N. aromaticivorans* F199 (65% id; 82% sim); S. sp. SKA58 (56% id; 77% sim); *S. wittichii* RW1 (36% id; 56% sim)

Lpl762: *S. japonicum* UT26 (73% id; 86% sim); *S.* sp. SKA58 (59% id; 76% sim); *N. aromaticivorans* F199 (51% id; 70% sim); *S. wittichii* RW1 (34% id; 52% sim)

Lpl773: *S. japonicum* UT26 (68% id; 80% sim); *N. aromaticivorans* F199 (51% id; 70% sim); *S.* sp. SKA58 (50% id; 66% sim)

Lpl783: Only in *S. japonicum* UT26 (65% id; 76% sim)

FIG S15 Lpl ORFs coding for glycosyl transferases and 4 subunits of an ABC-type polysaccharide transport system (A) and their homology with other sphingomonads (B). A close homologue of the hypothetical glycosyl transferase Lpl783 was found in *S. japonicum* UT26, whereas only very distantly related homologues of glycosyl transferases Lpl706, Lpl720 and Lpl731 can be found among sphingomonads.

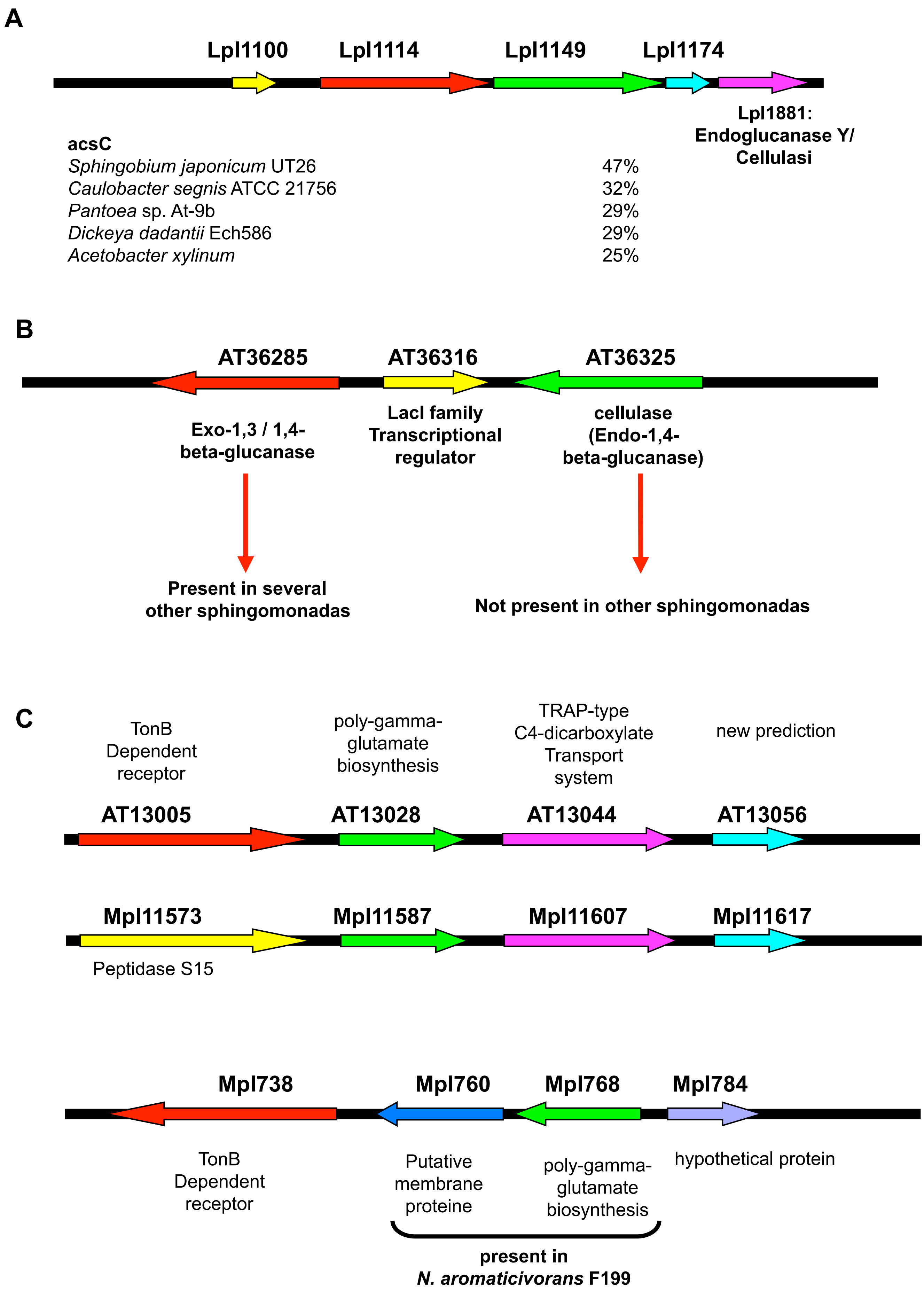
A
Lpl1100
Lpl1114
Lpl1149
Lpl1174
Lpl1881: Endoglucanase Y/ Cellulasi
acsC
Sphingobium japonicum UT26 47%
Caulobacter segnis ATCC 21756 32%
Pantoea sp. At-9b 29%
Dickeya dadantii Ech586 29%
Acetobacter xylinum 25%
B
AT36285
AT36316
AT36325
Exo-1,3 / 1,4-beta-glucanase
LacI family Transcriptional regulator
cellulase (Endo-1,4-beta-glucanase)
Present in several other sphingomonadas
Not present in other sphingomonadas
C
TonB Dependent receptor
poly-gamma-glutamate biosynthesis
TRAP-type C4-dicarboxylate Transport system
new prediction
AT13005
AT13028
AT13044
AT13056
Mpl11573
Mpl11587
Mpl11607
Mpl11617
Peptidase S15
Mpl738
Mpl760
Mpl768
Mpl784
TonB Dependent receptor
Putative membrane proteine
poly-gamma-glutamate biosynthesis
hypothetical protein
present in N. aromaticivorans F199

FIG S16 Lpl ORFs coding for a cellulose synthase and a hypothetical cellulose (A). Chromosome ORF coding for a hypothetical cellulase (AT36325) near to an ORF coding for an exo-1,3/1,4-beta-glucanase (B). ORFs coding for γ-PGA polymerases (C). One of these ORFs (Mpl768) is adjacent to an ORF coding for a hypothetical membrane protein. The same couple of ORFs is present in the genome of *N. aromaticivorans* F199 but not in other sphingomonads. The other two ORFs are part of two similar clusters of three ORFs located on the chromosome and megaplasmid. Interestingly, this cluster is not present in other sphingomonads.
