# Supplementary material for: Complete sequencing of Novosphingobium sp. PP1Y reveals a biotechnologically meaningful metabolic pattern
Source: BMC Genomics. 2014 May 19;15(1):384. doi: 10.1186/1471-2164-15-384 (PMC4059872; doi:10.1186/1471-2164-15-384)
Supplement: Supplementary file 2 — Additional file 2: Supplementary Tables and Figures. (PDF 70 KB) [file 12864_2013_6112_MOESM2_ESM.pdf]

Additional file 2

Complete sequencing of *Novosphingobium* sp. PP1Y reveals a biotechnologically meaningful metabolic pattern

Valeria D'Argenio[1,2,*], Eugenio Notomista[3,*], Mauro Petrillo[1,2], Piergiuseppe Cantiello[1], Valeria Cafaro[3], Viviana Izzo[4], Barbara Naso[1,2], Luca Cozzuto[1], Lorenzo Durante[3], Luca Troncone[3], Giovanni Paolella[1,2], Francesco Salvatore[1,5,§], Alberto Di Donato[3]

*These authors contributed equally to the work

[§]Corresponding author

Francesco Salvatore E-mail: salvator@unina.it

[1]CEINGE-Biotecnologie Avanzate, Napoli, Italy.

[2]Dipartimento di Medicina Molecolare e Biotecnologie Mediche, Università di Napoli Federico II;

[3]Dipartimento di Biologia, Università di Napoli Federico II;

[4]Dipartimento di Medicina e Chirurgia, Università degli Studi di Salerno;

[5]IRCCS-Fondazione SDN, Naples, Italy

Supplementary Results and Discussion

Genetic basis of aromatic hydrocarbons degradation

Five potential open reading frames (ORFs) for cytochrome P450 monooxygenases (MO) were identified. Most sphingomonads have from 1 to 5 ORFs for cytochrome P450-MO, whereas strains RW1 and F199 have 15 cytochrome P450-MO ORFs. These enzymes are versatile MO that can be involved in growth substrate activation, detoxification of toxic compounds and production of secondary metabolites. Cytochrome P450-MO can catalyze also the demethylation of aromatic metoxy or (di)methylamino groups [1]. Interestingly, PP1Y ORFs AT30146 and the adjacent AT30157 code for a cytochrome P450-MO and an arylamine-N-acetyltransferase, respectively. Together these enzymes could catalyze the detoxification of the toxic and mutagenic (di)methyl-arylamines to acetanilides.

Three potential ORFs for Baeyer Villiger monooxygenases/ketone monooxygenases (BVMO) were also identified. These enzymes, by catalyzing the monooxygenation of ketones to esters, perform a crucial role in the degradation of several xenobiotics [2]. The genomes of strains F199, L-1 and RW1 code for 11, 7 and 4 BVMOs respectively (Supplementary Figure S9). As in the case of ring cleavage dioxygenases (RCDs), each strain may have independently acquired a specific set of isoforms by horizontal transfer and in some cases by duplication (e.g., proteins 4343338 and 334343418 from strain L-1). However, in this case, it is difficult to hypothesize the substrate specificity from the phylogenetic tree, at least for PP1Y BVMOs.

Molybdopteryn-dependent oxygenases (MoMOs) are peculiar enzymes that use a molybdenum containing cofactor as redox center. These enzymes can catalyze complex reactions such as the monooxygenation of nitrogen-containing heterocyclic compounds (like xantine, quinoline and isoquinoline), the oxidation of carbon monoxide to CO2, and also the oxidation of several aldehydes [3]. Five PP1Y ORFs code for hypothetical MoMOs. The tree of MoMOs (Supplementary Figure S10) like that of RCDs and BVMOs, shows a heterogeneity among sphingomonads probably due to independent horizontal gene transfer events. As MoMOs are frequently involved in the catabolism of (iso)quinolines, we tested quinoline, isoquinoline and several methylquinolines dissolved in paraffin phases as the sole carbon and energy source as previously described for polycyclic aromatic hydrocarhons (PAHs) [4]. Strain PP1Y is not able to use isoquinoline or 3-methylquinoline, whereas it is able to grow, albeit slowly, using quinoline, 2-, 4-, 6-, 7- and 8-methylquinoline as the sole carbon and energy source. Interestingly, these quinolines induce excessive production of extracellular material similar to that induced by pyrene

and the heterocyclic compounds dibenzofuran, dibenzothiophene and carbazole [4]. The range of methylquinolines that can be used as carbon source suggests that at least two distinct initial hydroxylation events could take place in strain PP1Y (Supplementary Figure S11), however, at present it is not possible to hypothesize which MoMOs are involved.

Genetic basis of extracellular polymer secretion (EPS) and biofilm formation

Strain PP1Y shows a very complex "social" behavior; in fact, it is able to form different types of multicellular amorphous aggregates and ordered biofilm. PP1Y cultures, in both rich and minimal media, contain variable amounts of amorphous flocks and often, in the presence of hydrophobic polymers like polypropylene and polystyrene, they form a biofilm on the tube wall. When growing on diesel oil, the strain also forms a biofilm at the surface of diesel oil: in static cultures it colonizes the entire interface, whereas, during orbital shaking, it stabilizes the oil/water emulsion by coating the oil drops with biofilm. However, the chemical composition of growth media can influence the behavior of the strain. For example, phosphates [4] and glutamate (unpublished results) stimulate the formation of amorphous flocks, whereas oil drops and phases are colonized only if they contain specific aromatic compounds like pyrene and heterocyclic aromatic compounds [4].

Cells constitutively release a "hormone" at a constant rate; this "hormone" accumulates in the medium and, above a threshold concentration, it binds to a cell receptor activating the transcription of a specific set of genes. Among gram-negative strains, the most common hormones are the acyl-homoserine lactones that are synthesized by the protein LuxI and bind the LuxR receptor [5]. The PP1Y genome contains two copies, on the Chr and Lpl, of three ORFs coding for LuxR, LuxI and a phytanoyl-CoA dioxygenase like protein (Supplementary Figure S13A). Phytanoyl-CoA dioxygenases catalyze the hydroxylation of phytane, a branched long chain fatty acids [6], and could be involved in the synthesis of the acyl-moiety of the acyl-homoserine lactones. Even in this case, each sphingomonad has its own peculiar set of ORFs/proteins. For example, strain F199 lacks both LuxI and phytanoyl-CoA dioxygenase, RW1 has a single isoform of each protein, US6-1 has a single isoform of LuxI and three of phytanoyl-CoA dioxygenase (Supplementary Figure S13B and C). The chromosomally coded LuxI and phytanoyl-CoA dioxygenase of PP1Y (ORFs AT16460 and AT16449) are closely related to two sequences from strain US6-1, whereas the two proteins coded by ORFs on Lpl (Lpl262 and Lpl265) are closely related to the proteins of *S. alaskensis* RB2256 (Supplementary Figure S13B and C).

Several sphingomonads produce soluble acidic polysaccharides known as sphingans, which could have a wide spectrum of industrial applications. Examples are gellan, diutan and welan [7,8]. All these polysaccharides show the same repeated tetrasaccharidic unit but differ in the nature of the ramifications and/or modifications of the common backbone. The genes necessary for the synthesis of gellan, diutan and sphingans S-88 are known and form three very similar clusters of ORFs in strains *Sphingomonas elodea*, *Sphingomonas* sp. ATCC 53159 and *Sphingomonas* ATCC 31554 [7]. A similar cluster is not present in the PP1Y genome.

The cellulose synthase from *Acetobacter xylinum* is a prototype among the bacterial cellulose synthases [9]. (Saxena et al., 1994). These membrane enzymes synthesize, secrete and deposit cellulose fibrils outside the cell [9].

The machinery for the secretion of γ-PGA has been studied prevalently in Gram-positive strains and includes a membrane polymerase and a few accessory subunits that simultaneously synthesize and export the polymer [10]. In some cases, a transferase catalyzes the transfer of the chain to an acceptor on the cell surface (for example, a protein) thus avoiding the release in the medium [10].

References

1. Armengaud J and Timmis KN: **The reductase RedA2 of the multicomponent dioxin dioxygenase system of Sphingomonas sp. RW1 is related to class-I cytochrome P450-type reductases.** *Eur J Biochem* 1998, **253**:437–444.
2. Leisch H, Shi R, Grosse S, Morley K, Bergeron H, Cygler M, Iwaki H, Hasegawa Y, Lau PC: **Cloning, Baeyer-Villiger Biooxidations, and Structures of the Camphor Pathway 2-Oxo-Δ3-4,5,5-Trimethylcyclopentenylacetyl-Coenzyme A Monooxygenase of Pseudomonas putida ATCC 17453.** *Appl Environ Microbiol* 2012, **78**:2200–2212.
3. Hille R: **Molybdenum-containing hydroxylases.** *Arch Biochem Biophys* 2005, **433**:107–116.
4. Notomista E, Pennacchio F, Cafaro V, Smaldone G, Izzo V, Troncone L, Varcamonti M, Di Donato A: **The marine isolate Novosphingobium sp. PP1Y shows specific adaptation to use the aromatic fraction of fuels as the sole carbon and energy source.** *Microb Ecol* 2011, **61**:582-594.
5. Li YH and Tian X: **Quorum Sensing and Bacterial Social Interactions in Biofilms.** *Sensors* 2012, **12**:2519–2538.
6. Hao Y, Winans SC, Glick BR, Charles TC: **Identification and characterization of new LuxR/LuxI-type quorum sensing systems from metagenomic libraries**. *Environ Microbiol* 2010, **12**:105–117.
7. Gai Z, Wang X, Zhang X, Su F, Wang X, Tang H, Tai C, Tao F, Ma C, Xu P: **Genome sequence of Sphingomonas elodea ATCC 31461, a highly productive industrial strain of gellan gum.** *J Bacteriol* 2001, **193**:7015–7016.
8. Fialho AM, Moreira LM, Granja AT, Popescu AO, Hoffmann K, Sá-Correia I: **Occurrence, production, and applications of gellan: current state and perspectives.** *Appl Microbiol Biotechnol* 2008, **79**:889-900.
9. Saxena IM, Kudlicka K, Okuda K, R M Brown RM: **Characterization of genes in the cellulose-synthesizing operon (acs operon) of Acetobacter xylinum: implications for cellulose crystallization.** *J Bacteriol* 1994, **176**:5735–5752.
10. Lemon KP, Earl AM, Vlamakis HC, Aguilar C, Kolter R: **Biofilm Development with an Emphasis on Bacillus subtilis.** *Curr Top Microbiol Immunol* 2008, **322**:1–16.
